# Three New Triterpene Glycosides from the Roots of *Deutzia* x *Hybrida* “Strawberry Fields” (Hydrangeaceae)

**DOI:** 10.3390/molecules29235781

**Published:** 2024-12-06

**Authors:** Efstathia Karachaliou, David Pertuit, Antoine Bruguière, Marie-José Penouilh, Michel Picquet, Christine Belloir, Loïc Briand, Anne-Claire Mitaine-Offer

**Affiliations:** 1 Centre des Sciences du Goût et de l’Alimentation, CNRS, INRAE, Institut Agro, Université de Bourgogne, 21000 Dijon, CEDEX, France; efstathia.karachaliou@u-bourgogne.fr (E.K.); david.pertuit@u-bourgogne.fr (D.P.); antoine.bruguiere@u-bourgogne.fr (A.B.); christine.belloir@inrae.fr (C.B.); loic.briand@inrae.fr (L.B.); 2 Institut de Chimie Moléculaire de l’Université de Bourgogne (UMR 6302), CNRS, Université de Bourgogne, 9 Av. Alain Savary, BP 47870, 21078 Dijon, CEDEX, France; marie-jose.penouilh@u-bourgogne.fr (M.-J.P.); michel.picquet@u-bourgogne.fr (M.P.)

**Keywords:** *Deutzia* x *hybrida* “Strawberry Fields”, Hydrangeaceae, triterpene glycosides, sweet taste, TAS1R2/TAS1R3

## Abstract

Three new triterpene glycosides were isolated from *Deutzia* x *hybrida* “Strawberry Fields” cultivar via aqueous–ethanolic extraction of the roots, including one derivative of sumaresinolic acid and two of echinocystic acid: 3-*O*-β-D-glucuronopyranosylsumaresinolic acid 28-*O*-β-D-xylopyranosyl-(1→4)-α-L-rhamnopyranosyl-(1→2)-α-L-arabinopyranosyl ester, 3-*O*-β-D-glucuronopyranosylechinocystic acid 28-*O*-β-D-xylopyranosyl-(1→4)-α-L-rhamnopyranosyl-(1→2)-α-L-arabinopyranosyl ester, and 3-*O*-α-L-arabinopyranosyl-(1→3)-β-D-glucuronopyranosylechinocystic acid 28-O-β-D-xylopyranosyl-(1→4)-α-L-rhamnopyranosyl-(1→2)-α-L-arabinopyranosyl ester. As none of the isolated saponins were previously documented in the literature, their structural elucidation required extensive 1D and homo- and heteronuclear 2D NMR spectroscopy, as well as mass spectrometry analysis. All three glycosides were tested for their stimulatory activity of the sweet taste receptor TAS1R2/TAS1R3. It is the first chemical and biological investigation of *Deutzia* x *hybrida* “Strawberry Fields” as well as the first report of sumaresinolic acid glycosides in *Deutzia* genus.

## 1. Introduction

*Deutzia* Thunb. is a genus of the Hydrangeaceae family, among the seven families belonging to the Cornales order [1]. The 60 species of this genus are widespread in warm temperate climate, from Southeast Asia to the Philippines, West and South of North America, and Central America. *Deutzia* is phylogenetically most closely related to *Philadelphus*, and both were formerly considered to belong to the family *Saxifragaceae*. However, more recent chemotaxonomic classifications consider these, and other woody genera, to belong to the *Hydrangeaceae* [2]. South-West China has been identified as the centre of diversity for the genus, based on its rich species diversity. The *Deutzia* species hold an economic significance as ornamental plants, with hybrids such as *D.* x *hybrida,* from *D. longifolia* and *D. discolor*. In garden centres, many cultivars can be found such as the “Strawberry Fields”, a deciduous shrub with pink flowers bordered with white [3]. Its dark green foliage adds to its visual appeal, despite its lack of fragrance and attraction for the pollinators. This variety does not have remarkable bark characteristics or any known toxicity.

From a phytochemistry point of view, the literature data are relatively scarce for saponins from *Deutzia* but one study described the identification of two echinocystic acid glycosides, Deutzicoside A and B, from *D. corymbosa* [4]. The aglycon part, echinocystic acid, was found in the literature to possess many biological activities such as antiviral, anti-inflammatory, and antioxidant. Studies show it enhances spatial memory in elderly mice by regulating the JNK signalling pathway and promoting neurite growth [5]. Moreover, the hydroxylation of this oleanane-type aglycon, and the 3-*O*-heterosidic linkage with a glucuronic acid moiety, may represent a good requirement for the activation of the sweet taste receptor TAS1R2/TAS1R3 according to our previous research [6].

So, we choose to achieve the phytochemical study of a well-known ornamental plant *D.* x *hybrida,* “Strawberry Fields”, and the isolation of three glycosides not previously described in the literature, including one derivative of sumaresinolic acid (**1**) and two of echinocystic acid (**2**,**3**). All three compounds were tested for their stimulatory activity on the sweet taste receptor TAS1R2/TAS1R3.

## 2. Results and Discussion

**Figure 1:**
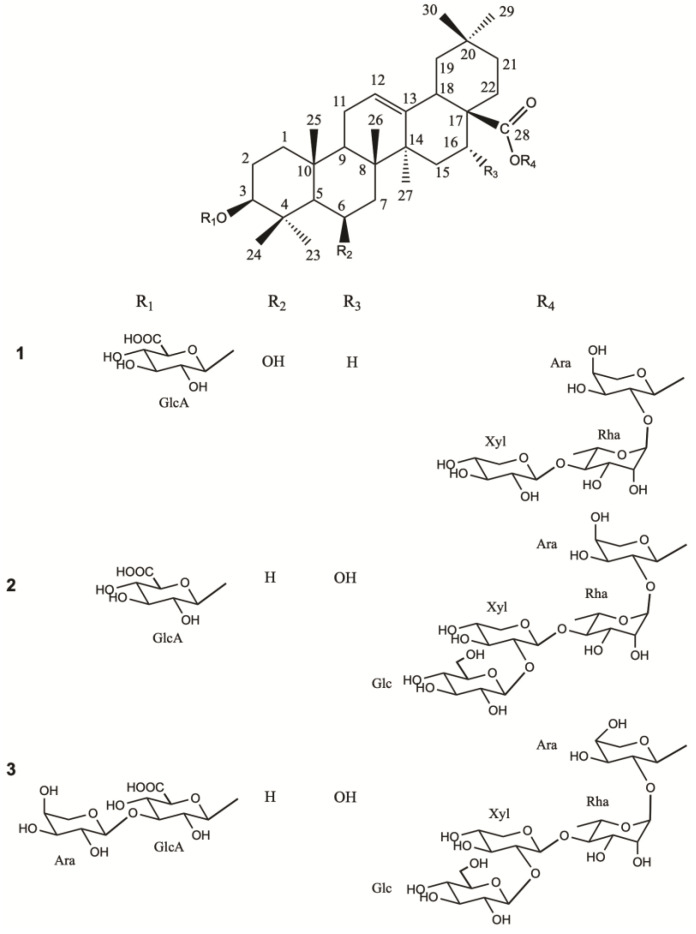
Structures of saponins **1**–**3**.

The aqueous–ethanolic (35:75) extract of powdered dried roots of *D.* x *hybrida* “Strawberry Fields” was subjected to multiple chromatographic steps (VLC, flash column, MPLC, see the Section 3.1) to separate, isolate, and purify three new saponins (**1**–**3**) (Figure 1).

The structural elucidation of the three triterpene glycosides **1**–**3** was achieved using mainly homo- and heteronuclear 2D NMR experiments and electrospray mass spectrometry in positive-ion mode. For the osidic part of these molecules, the analysis of the COSY, TOCSY, ROESY, HSQC, and HMBC spectra revealed the presence of glucuronic acid, arabinose, rhamnose and xylose for **1**, and glucuronic acid, arabinose, rhamnose, xylose, and glucose for **2** and **3**. Their absolute configurations were determined to be D for glucuronic acid (GlcA), glucose (Glc), and xylose (Xyl), and L for arabinose (Ara) and rhamnose (Rha) (see the Section 3.1). The relatively large ^3^*J*_H-1, H-2_ values of the GlcA, Glc, Xyl, and Ara (4.0–7.8 Hz) indicated a β anomeric orientation for GlcA, Glc, and Xyl, and an α anomeric orientation for Ara [7,8]. For the Rha moieties, large ^1^*J*_H-1, C-1_ values at 166–168 Hz confirmed that the anomeric protons were in an equatorial α-pyranoid form [7]. The structural analysis of the aglycons and the identification of the linkages with the oligosaccharidic chains for each compound are described below.

**Figure 2:**
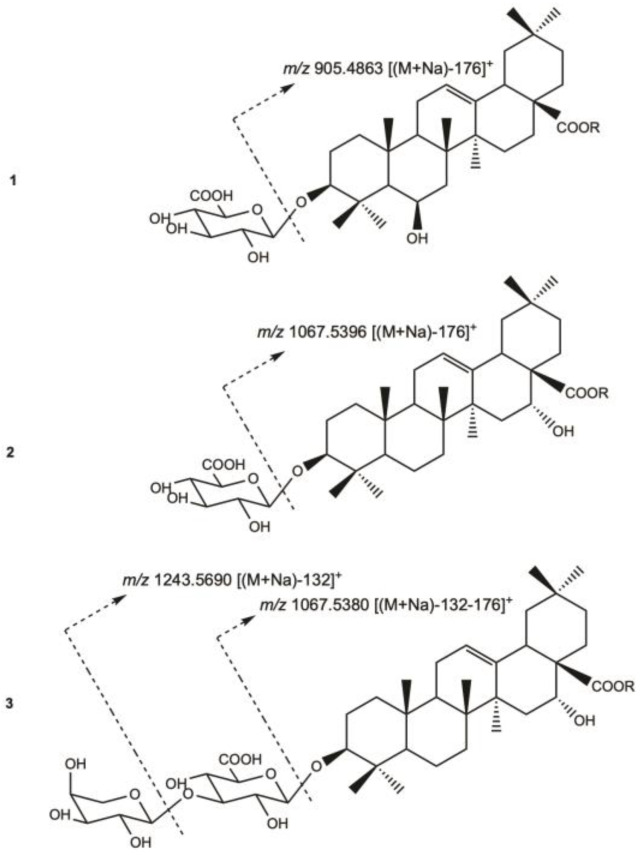
Fragment ions (*m*/*z*) of pseudo-molecular ion peaks for **1**–**3** (HR-ESIMS positive-ion mode).

For compound **1**, the HR-ESIMS (positive-ion mode) spectrum showed a pseudo-molecular ion peak at *m*/*z* 1081.5177 [(M+Na)]^+^, indicating a molecular weight of 1058 and a molecular formula of C_52_H_82_O_22_. A fragmentation peak at *m*/*z* 905.4863 [(M+Na)-176]^+^ indicated the loss of one uronic acid (Figure 2).

**Figure 3:**
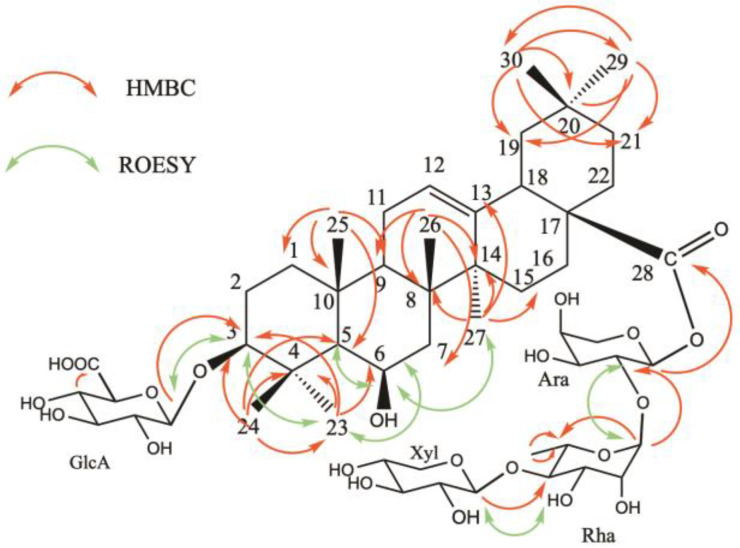
Key HMBC and ROESY correlations for compound **1**.

For the aglycon part, the HSQC spectrum displayed signals for seven angular methyl groups of a triterpene skeleton at *δ*_C_/*δ*_H_ 27.9/1.13 (s) (CH_3_-23), 18.0/1.22 (s) (CH_3_-24), 17.1/1.31 (s) (CH_3_-25), 18.6/1.05 (s) (CH_3_-26), 26.2/1.12 (s) (CH_3_-27), 33.2/0.90 (s) (CH_3_-29), 23.6/0.94 (s) (CH_3_-30), signals of an ethylene bond at *δ*_C_/*δ*_H_ 124.0/5.33 (CH-12), an ester function at 177.6, and two secondary alcoholic functions at *δ*_C_/*δ*_H_ 91.0/3.10 (dd, *J* = 4.5, 11.7 Hz) and 68.4/4.51 ppm (Table 1, Supplementary Materials). The HMBC correlations between *δ*_H_ 1.13 (s) (H_3_-23) and 1.22 (s) (H_3_-24), and *δ*_C_ 91.0 revealed the location of the first hydroxyl group at C-3 position. Moreover, the HMBC cross-peak at *δ*_H/_*δ*_C_ 0.76 (d, *J* = 6.8 Hz) (H-5)/68.4 proved the location of the second hydroxyl function at C-6 position. The configurations of C-3 and C-6 were determined by the correlations observed in the ROESY spectrum between, for example, H_3_-23 α-equatorial, H-3 α-axial, and H-6 α-equatorial (Figure 3). The total assignment of the carbons and protons allowed the identification of the aglycon as 3β,6β-dihydroxyolean-12-en-28-oic acid, named sumaresinolic acid [9,10] (Table 1). The chemical shifts at *δ*_C_ 91.0 ppm for C-3 and 177.6 for C-28, suggested a bidesmosidic structure with an *O*-heterosidic linkage at the C-3 position, and an ester linkage at C-28.

For the osidic residues, the ^1^H NMR spectrum of compound **1** showed four anomeric proton signals at *δ*_H_ 5.64 (d, *J* = 4.0 Hz), 5.10 (br s), 4.50 (d, *J* = 7.8 Hz), and 4.37 (d, *J* = 7.8 Hz), which showed correlations in the HSQC spectrum with their respective anomeric carbons at *δ*_C_ 93.6, 101.1, 106.4, and 106.6. The protons and carbons signals of the sugars were identified using 2D NMR experiments, primarily starting with the TOCSY experiment. Additionally, the COSY experiment provided key information regarding the positions of geminal protons in the sugar moieties, at *δ*_H_/*δ*_H_ 5.64 (Ara H-1)/3.83 (Ara H-2), 5.10 (Rha H-1)/3.89 (Rha H-2), 4.50 (Xyl H-1)/3.21 (Xyl H-2), 4.37 (GlcA H-1)/3.25 (GlcA H-2), 3.91 (Ara H-5a)/3.50 (Ara H-5b), 3.86 (Xyl H-5a)/3.18 (Xyl H-5b), and 3.75 (Rha H-5)/1.29 (Rha CH_3_-6). This analysis resulted in the identification of GlcA, Ara, Rha, and Xyl units (Table 2). The β-D-glucuronopyranosyl moiety was determined to be linked at the C-3 position of the aglycone by an HMBC correlation between δ_H_ 4.37 (GlcA H-1) and δ_C_ 91.0 (C-3), and the ROESY correlation at *δ*_H_/*δ*_H_ 4.37 (GlcA H-1)/3.10 (H-3). The shielded value of the Ara C-1 at 93.8 ppm and the HMBC cross-peak at δ_H_/δ_C_ 5.64 (Ara H-1)/177.6 (C-28), suggested the ester linkage at C-28. Furthermore, the HMBC spectrum of compound **1** displayed correlations at *δ*_H_/*δ*_C_ 5.10 (Rha H-1)/75.3 (Ara C-2) and 4.50 (Xyl H-1)/83.1 (Rha C-4), which suggested the structure of the oligosaccharidic part as 28-*O*-β-D-xylopyranosyl-(1→4)-α-L-rhamnopyranosyl-(1→2)-α-L-arabinopyranosyl (Figure 3). This was confirmed by the ROESY cross-peaks at *δ*_H_/*δ*_H_ 5.10 (Rha H-1)/3.83 (Ara H-2) and 4.50 (Xyl H-1)/3.56 (Rha H-4). To conclude, the structure of compound **1** was established as 3-*O*-β-D-glucuronopyranosylsumaresinolic acid 28-*O*-β-D-xylopyranosyl-(1→4)-α-L-rhamnopyranosyl-(1→2)-α-L-arabinopyranosyl ester.

For compound **2**, the HR-ESIMS (positive-ion mode) spectrum showed a pseudo-molecular ion peak at *m*/*z* 1243.5719 [(M+Na)]^+^, indicating a molecular weight of 1220 and a molecular formula of C_58_H_92_O_27_, with an additional hexosyl moiety of 162 amu compared to compound **1**. The pseudomolecular ion showed a fragmentation peaks at *m*/*z* 1067.5396 [(M+Na)-176]^+^, indicating the loss of a uronic acid.

In the HSQC spectrum of the aglycon part of compound **2** (Table 1), seven typical angular methyl groups were found at *δ*_C_/*δ*_H_ 28.3/1.06 (s) (CH_3_-23), 16.7/0.85 (s) (CH_3_-24), 16.0/0.96 (s) (CH_3_-25), 17.5/0.77 (s) (CH_3_-26), 26.8/1.36 (s) (CH_3_-27), 33.3/0.88 (s) (CH_3_-29), 24.8/0.96 (s) (CH_3_-30), as well as signals of an ethylene bond at *δ*_C_/*δ*_H_ 123.5/5.34 (CH-12), an ester function at 175.6, and two secondary alcoholic functions at *δ*_C_/*δ*_H_ 91.0/3.17 and 74.3/4.49 ppm (Table 1). All these signals were in accordance with a 3β-hydroxyolean-12-en-28-oic acid skeleton with an additional hydroxyl function. Its location at the C-16 position was determined using the HMBC spectra, with cross-peaks at *δ*_H_/*δ*_C_ 4.49 (H-16)/42.4 (C-14) and 1.77, 1.91 (H_2_-22)/74.3 (C-16). Its configuration was established by the ROESY correlation at *δ*_H_/*δ*_H_ H_3_-26 β-axial at 0.77 and H-16 β-equatorial at 4.49 ppm. Thus, the aglycon was identified as 3β,16α-dihydroxyolean-12-en-28-oic acid named echinocystic acid [4,11]. The chemical shifts at *δ*_C_ 91.0 for C-3 and 175.6 ppm for C-28 suggested a bidesmosidic structure with an *O*-heterosidic linkage at the C-3 position and an ester linkage at C-28.

For the osidic part of the molecule, the ^1^H NMR spectrum of compound **2** showed five anomeric proton signals at *δ*_H_ 5.54 (d, *J* = 4.3 Hz), 5.45 (br s), 4.55 (d, *J* = 7.7 Hz), 4.41 (d, *J* = 7.7 Hz), and 4.38 (d, *J* = 7.7 Hz), which showed correlations in the HSQC spectrum with their respective anomeric carbons *δ*_C_ 93.8, 100.1, 106.0, 106.9, and 106.6. The identification of GlcA, Ara, Rha, Xyl, and Glc moieties was obtained by 2D NMR analysis (Table 2). The 3-*O*-β-D-glucuronopyranosyl linkage was determined according to an HMBC correlation between δ_H_ 4.38 (GlcA H-1) and δ_C_ 91.0 ppm (C-3), and the ROESY correlation at *δ*_H_/*δ*_H_ 4.38 (GlcA H-1)/3.17 (H-3). The 28-*O*-β-D-xylopyranosyl-(1→4)-α-L-rhamnopyranosyl-(1→2)-α-L-arabinopyranosyl sequence was elucidated using the HMBC spectrum with cross-peaks at δ_H_ 5.54 (Ara H-1)/δ_C_ 175.6 (C-28), 5.45 (Rha H-1)/75.8 (Ara C-2), and 4.41 (Xyl H-1)/81.6 (Rha C-4). An additional terminal β-D-glucopyranosyl moiety was found based on the 4.55 (Glc H-1)/82.6 (Xyl C-2) correlation. The linkages were ensured by the ROESY cross-peaks at *δ*_H_/*δ*_H_ 5.45 (Rha H-1)/3.77 (Ara H-2), 4.41 (Xyl H-1)/3.94 (Rha H-4), and 4.55 (Glc H-1)/3.58 (Xyl H-2). Thus, the structure of compound **2** was elucidated as 3-*O*-β-D-glucuronopyranosylechinocystic acid 28-*O*-β-D-glucopyranosyl-(1→2)-β-D-xylopyranosyl-(1→4)-α-L-rhamnopyranosyl-(1→2)-α-L-arabinopyranosyl ester.

For compound **3**, the HR-ESIMS (positive-ion mode) spectrum showed a pseudo-molecular ion peak at *m*/*z* 1375.6121 [(M+Na)]^+^, indicating a molecular weight of 1352 and a molecular formula of C_63_H_100_O_31_, with an additional 132 amu compared to compound **2**, corresponding to a pentosyl moiety. The HR-ESIMS/MS (positive-ion mode) of the pseudomolecular ion showed fragmentation peaks at *m*/*z* 1243.5690 [(M+Na)-132]^+^, and 1067.5380 [(M+Na)-176]^+^, indicating a successive loss of a pentosyl and a uronic acid moieties. The NMR signals of compound **3** are almost superimposable with those of compound **2**, with a common sequence of 3-*O*-β-D-glucuronopyranosylechinocystic acid 28-*O*-β-D-glucopyranosyl-(1→2)-β-D-xylopyranosyl-(1→4)-α-L-rhamnopyranosyl-(1→2)-α-L-arabinopyranosyl ester, with an additional arabinopyranosyl moiety. Moreover, the HMBC correlation at *δ*_H_/*δ*_C_ 4.32 (Ara II H-1)/80.8 (GlcA C-3) and the ROESY correlation at 4.32 (Ara II H-1)/3.70 (GlcA H-3), suggested a substitution of the GlcA-3 position by a terminal arabinopyranosyl residue. The structure of compound **3** was established as 3-*O*-α-L-arabinopyranosyl-(1→3)-β-D-glucuronopyranosylechinocystic acid 28-*O*-β-D-glucopyranosyl-(1→2)-β-D-xylopyranosyl-(1→4)-α-L-rhamnopyranosyl-(1→2)-α-L-arabinopyranosyl ester.

**Figure 4:**
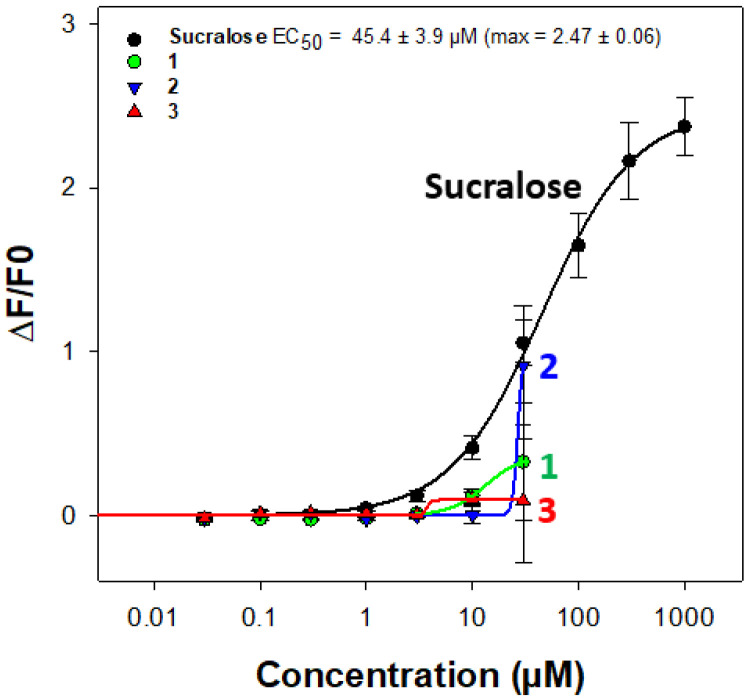
Diagram of TAS1R2/TAS1R3 activity response for sucralose (black curve) and compounds **1**–**3** (respectively green curve for **1**, blue for **2**, and red for **3**). The x-axis shows the concentration (µM). The y-axis shows the relative changes in fluorescence (ΔF/F0). The ΔF/F0 values are expressed as the mean ± sem (n = 6). EC50: half maximal effective concentration. Max: maximal amplitude of fluorescence signal.

The search for sweeteners to replace the use of sugar in food is still relevant. Among the naturally occurring molecules, 85 plant-derived sweet compounds of 19 major structural types are known [12], mainly triterpenoid glycosides, as glycyrrhizin from licorice [13]. To evaluate the sweetness of the natural compounds, the stimulation of the human taste heterodimer receptor TAS1R2/TAS1R3 is studied [14]. So, with sucralose as a reference, this experiment was achieved with the triterpene glycosides **1**–**3**. Unfortunately, the results indicate that these compounds do not activate the receptor. Indeed, even if an increase in intracellular calcium levels is measured for the three compounds at 30 µM, the test substances elicited significant artificial calcium responses in the absence of TAS1R2/TAS1R3 in mock-transfected cells at this same concentration. Furthermore, as the compounds were only available in limited quantities, this prevented us from testing higher concentrations to measure potentially greater differences between signal-to-noise ratio and establishing a dose–response relationship to deduce EC50 values (Figure 4).

As we have previously reported, about the structure–activity relationships, the hydroxylation of an oleanane-type aglycon, as for sumaresinolic and echinocystic acid, and the 3-*O*-heterosidic linkage with a glucuronic acid moiety, should represent a good requirement for the activation of the sweet taste receptor [6]. But, in a recent publication [15], mono- and bidesmosidic saponins were evaluated, and none of the bidesmosides were active. This is supported by the results obtained with the bidesmosides of *D.* x *hybrida* “Strawberry Fields”.

In conclusion, the structural elucidation of three new compounds (**1**–**3**) isolated from *D.* x *hybrida* “Strawberry Fields” was successfully achieved using extensive 2D NMR and ESIMS analyses. These compounds were identified as bidesmosides, with sumaresinolic acid as the aglycon for compound **1** and echinocystic acid for compounds **2** and **3**. The first structural differences among the compounds are the hydroxyl group positions in the aglycons and the addition of sugar units in the glycosidic chains: compound **2** contains an additional glucopyranosyl unit compared to compound **1**, and compound **3** contains an additional arabinopyranosyl unit compared to compound **2**. Their structures add interesting variability to the scarce literature about saponins from *Deutzia* genus and allow to confirm that the bidesmosidic structure does not activate the TAS1R2/TAS1R3 receptor.

## 3. Materials and Methods

### 3.1. General Experimental Procedures

Optical rotations were recorded on an AA-OR automatic polarimeter. Melting points were determined on a Wagner Munz (Munich, Germany) Kofler Hot Bench and are uncorrected. The 1D and 2D NMR experiments (^1^H, COSY, TOCSY, ROESY, edited ^1^H-^13^C HSQC, and ^1^H-^13^C HMBC) were performed on a Bruker (Billerica, MA, USA**)** Avance III HD 600 spectrometer equipped with an ATMA BBOF 5 mm Prodigy Cryoprobe (Bruker) with a z-axis gradient coil operating at 600 MHz (^1^H). Spectra were acquired in 5 mm NMR tubes and all experiments were performed at 298K and run without spinning to avoid convection. All the samples were prepared in methanol-*d_4_* (CD_3_OD) with 0.1% trifluoroacetic acid (TFA). Chemical shifts on the δ scale (ppm) relative to tetramethylsilane were referenced internally with respect to the resonance of residual CH_3_OH (δ_H_ = 3.31 ppm and δ_C_ = 49.00 ppm). Coupling constants (J) were measured in Hz. ESIMS (positive-ion mode) was performed using an Orbitrap Fusion instrument (Thermo Fisher Scientific, Waltham, MA, USA) with a resolution of 120,000 and a mass accuracy of 4 ppm for the identification of molecular formulas. The samples were prepared using a resuspension solvent composed of 1 mL methanol/water (70:30) with 0.1% formic acid (FA). After dissolution, the sample was vortexed and then subjected to microfiltration using a 0.20 μm PTFE filter. Direct injection was carried out at a flow rate of 7 μL/min using a High-Flow needle, and the source and detector parameters were optimized according to the sample. A MARS 6 microwave apparatus (CEM Corporation, Matthews, NC, USA) was used for the extractions. The vacuum liquid chromatography (VLC) was performed using silica gel 60 (63–200 µm, Sigma-Aldrich, Saint-Louis, MO, USA) and RP-18 silica gel (40–60 µm, AIT France, Cormeilles-en-Parisis, France). Flash chromatography was performed using a CombiFlash Retrieve (Teledyne ISCO, Lincoln, NE, USA) with RediSep Rf normal phase silica gel columns (normal phase, 40–63 µm, 40 g, max pressure 300 psi/20.7 bar, cylinder volume 48 mL). Silica gel 60 (15–40 µm, Merck, Burlington, MA, USA) with a Gilson (Middleton, WI, USA) M 305 pump (25 SC head pump, M 805 manometric module), a Büchi (Villebon-Sur-Yvette, France) glass column (230 × 15 mm and 460 × 15 mm) and a Büchi glass precolumn (110 × 15 mm) were used for medium pressure liquid chromatography (MPLC). Thin-layer chromatography (TLC, Silicycle, Québec, QC, Canada) and high-performance thin-layer chromatography (HPTLC, Merck) were carried out on precoated silica gel plates 60 F_254_, solvent system CHCl_3_/MeOH/H_2_O/CH_3_COOH (60:32:7:1). The spray reagent used for saponin identification was vanillin reagent (1% vanillin in EtOH/H_2_SO_4_, 50:1). The purification process as well as the profile of crude extracts, have been controlled with TLC. HPTLC and HPLC were used to indicate the purity of the final products. HPLC was performed on a 1260 Agilent instrument, equipped with a degasser, a quaternary pump, an autosampler, a UV detector at 210 nm. Eluent: (A) 0.01% (*v*/*v*) aqueous trifluoroacetic acid and (B) acetonitrile, 1 mL/min. The gradient system was 10% B to 70% B in 30 min for compound **2**, and 25% B to 45% B in 45 min for compounds **1** and **3**. Each pure compound was injected (10 μL) and analyzed, to give *tr* = 24.88, 13.68, and 19.01 min for **1**, **2**, and **3**, respectively.

### 3.2. Plant Material

*Deutzia* x *hybrida* “Strawberry Fields” was purchased from Botanic^®^ (Quétigny, France) in July 2022. A voucher specimen was stored separately in the herbarium at the Laboratory of Pharmacognosy, Centre des Sciences du Goût et de l’Alimentation (CSGA), Dijon, France, under the identifier N°2022/07/11.

### 3.3. Extraction and Isolation

The roots of *Deutzia* x *hybrida* “Strawberry Fields” were collected (58.01 g) and left to dry in a dark place at room temperature and then pulverized into powder to yield 55.5 g. Afterward, it was subjected to three consecutive circles of Microwave-Assisted Extraction (MAE), each time with 440 mL of EtOH/H_2_O, 75:35. The microwave had been configured to operate at 220 W and 30 °C, by gradually increasing from room temperature to 30 °C over the course of 15 min, and then maintaining that level of temperature for an additional 30 min. After filtration and evaporation of the extracted solvent after each circle, the crude extract of 7.23 g (7.67% yield) was subjected to a first crude fractionation to eliminate free sugars, polysaccharides, tannins, and unwanted highly polar metabolites using a reversed phase VLC (RP-18 silica gel, gradient H_2_O/EtOH 100:0, 0:100, 1.2 L each corresponding to 3 column volumes). The ethanolic fraction of 3.57 g was divided into two parts and each part was fractionated with another reversed-phase VLC (RP-18 silica gel, gradient H_2_O/EtOH 100:0, 75:25, 50:50, 0:100, 300 mL each corresponding to 3 column volumes). The 75:25 and 50:50 H_2_O/EtOH fractions of the two VLCs were grouped (1.7 g) and subjected to flash chromatography two times (flow rate 40mL/min, isocratic solvent system CHCl_3_/MeOH/H_2_O, 60:32:7, 64 fractions, 10 mL/fraction), to afford a crude saponin mixture (129 mg). The latter was purified by normal phase MPLCs (silica gel 60, flow rate 2.5 mL/min, isocratic solvent system CHCl_3_/MeOH/H_2_O, 64:40:8, 120 fractions, 1.25 mL/min), yielding compounds **1** (20.4 mg), **2** (9.0 mg) and **3** (17.3 mg).

*3-O-β-D-glucuronopyranosylsumaresinolic acid 28-O-β-D-xylopyranosyl-(1→4)-α-L-rhamnopyranosyl-(1→2)-α-L-arabinopyranosyl ester* (**1**).

White, amorphous powder; [α]^25^_D_ −20 (c 0.1, H_2_O); Mp 211–209 °C; ^1^H and ^13^C NMR data [600 MHz and 150 MHz, methanol-*d*_4_ (CD_3_OD) with 0.1% trifluoroacetic acid (TFA)], see Table 1 and Table 2; HR-ESIMS (positive-ion mode) *m*/*z* 1081.5177 [M+Na]^+^ (Calcd for C_52_H_82_O_22_ Na, 1081.5190); TLC: R_f_ 0.40, greyish spot by spraying with vanillin-H_2_SO_4_ reagent.

*3-O-β-D-glucuronopyranosylechinocystic acid 28-O-β-D-glucopyranosyl-(1→2)-β-D-xylopyranosyl-(1→4)-α-L-rhamnopyranosyl-(1→2)-α-L-arabinopyranosyl ester* (**2**)

White, amorphous powder; [α]^25^_D_ −15 (c 0.1, H_2_O); Mp 228–226 °C; ^1^H and ^13^C NMR data [600 MHz and 150 MHz, methanol-*d*_4_ (CD_3_OD) with 0.1% trifluoroacetic acid (TFA)], see Table 1 and Table 2; HR-ESIMS (positive-ion mode) *m*/*z* 1243.5719 [M+Na]^+^ (Calcd for C_58_H_92_O_27_ Na, 1243.5718); TLC: R_f_ 0.20, greyish spot by spraying with vanillin-H_2_SO_4_ reagent.

*3-O-a-L-arabinopyranosyl-(1→3)-β-D-glucuronopyranosylechinocystic acid 28-O-β-D-glucopyranosyl-(1→2)-β-D-xylopyranosyl-(1→4)-α-L-rhamnopyranosyl-(1→2)-α-L-arabinopyranosyl ester* (**3**)

White, amorphous powder; [α]^25^_D_ −10 (c 0.1, H_2_O); Mp 242–240 °C; ^1^H and ^13^C NMR data [600 MHz and 150 MHz, methanol-*d*_4_ (CD_3_OD) with 0.1% trifluoroacetic acid (TFA)], see Table 1 and Table 2; HR-ESIMS (positive-ion mode) *m*/*z* 1375.6121 [M+Na]^+^ (Calcd for C_63_H_100_O_31_ Na, 1375.6141); TLC: R_f_ 0.12, greyish spot by spraying with vanillin-H_2_SO_4_ reagent.

### 3.4. Acid Hydrolysis and Absolute Configuration Determination

An aliquot (100 mg) of a fraction-rich saponins was hydrolyzed with 2N aqueous CF_3_COOH (25 mL) for 3 h at 95 °C. After extraction with CH_2_Cl_2_ (3 × 15 mL), the aqueous layer was evaporated to dryness with H_2_O until neutral to give the sugar residue (30 mg). Glucuronic acid, arabinose, rhamnose, xylose, and glucose were identified by comparison with authentic samples by TLC using CH_3_COOEt/CH_3_COOH/CH_3_OH/H_2_O (65:25:15:15). After purification of these sugars by prep-TLC in the same solvent, the optical rotation of each purified sugar was measured as follows: D-glucuronic acid: *R*_f_ = 0.24, [α]^25^_D_ + 15 (*c* 0.2, H_2_O), L-arabinose: *R*_f_ = 0.54, [α]^25^_D_ + 170 (*c* 0.2, H_2_O), L-rhamnose: *R*_f_ = 0.69, [α]^25^_D_ + 10 (*c* 0.2, H_2_O), D-xylose: *R*_f_ = 0.60, [α]^25^_D_ + 85 (*c* 0.2, H_2_O), and D-glucose: *R*_f_ = 0.50, [α]^25^_D_ + 110 (*c* 0.2, H_2_O).

### 3.5. TAS1R2/TAS1R3 Bioactivity Assay

A recent experimental strategy to identify new sweeteners or taste modulators was employed during this module, which involves the use of heterologous expression of TAS1R2/TAS1R3 and functional calcium imaging. So, sweet taste receptor activation was evaluated using HEK293T cells expressing TAS1R2/TAS1R3 and GCaMP6S, a chimeric protein that acts as a calcium biosensor, and derives from a circularly permuted green fluorescent protein. The cells were then transfected with TAS1R cDNAs and GCaMP6S using Fugene HD, along with the chimeric G protein subunit Gα16gust44. Negative controls included cells transfected only with the calcium indicator. Before stimulation, cells were washed with buffer C1 (130 mM NaCl, 5 mM KCl, 10 mM Hepes, 2 mM CaCl2, 5 mM sodium pyruvate, pH 7.4) [16]. All three compounds reported very good initial solubilization in DMSO at 10 mM, followed by subsequent dilution in loading buffer C1 with no precipitates. All compounds were tested up to a final concentration of 100 µM, followed by a refined range with a maximum of 30 µM, with sucralose as a positive control. Calcium mobilization was monitored after automatic injection of the three test substances with a Molecular Devices FlexStation 3 system (SoftMax Pro 5.4.6), to measure the sweet taste receptor activation. Figure 4 illustrates the fluorescent calcium response at different concentrations of saponins **1**, **2**, and **3**.

## Figures and Tables

**Table 1:** ^13^C and ^1^H NMR spectroscopic data of the aglycons of **1**–**3** in CD_3_OD (*δ* in ppm, *J* in Hz).

Position	1		2		3	
	*δ*_C_	*δ*_H_	*δ*_C_	*δ*_H_	*δ*_C_	*δ*_H_
1	41.5	0.97, 1.67	39.6	1.01, 1.63	39.6	1.00, 1.64
2	26.9	1.76 m, 1.84 m	26.8	1.70 m, 1.85 m	26.8	1.70 m, 1.85 m
3	91.0	3.10 dd (11.7, 4.5)	91.0	3.17	90.9	3.18
4	40.8	-	39.9	-	39.8	-
5	57.0	0.76 d (6.8)	56.9	0.79	56.9	0.79
6	68.4	4.51 m	19.1	1.40, 1.56 m	19.2	1.40, 1.57
7	39.5	0.99, 1.63	34.0	1.37, 1.51	33.9	1.39, 1.53
8	39.6	-	40.6	-	40.4	-
9	49.1	1.60	47.8	1.64	47.4	1.64
10	37.0	-	36.1	-	36.0	-
11	24.1	1.90, 1.90	24.4	1.90, 1.90	24.2	1.90, 1.90
12	124.0	5.33 br t (3.5)	123.5	5.34 br t (3.2)	123.4	5.34 br t (3.3)
13	143.8	-	144.4	-	144.2	-
14	43.1	-	42.4	-	42.3	-
15	28.8	1.15, 1.72	36.1	1.40, 1.76	36.1	1.41, 1.75
16	23.7	1.66, 2.01 td (13.9, 2.8)	74.3	4.49 m	74.3	4.49 m
17	46.2	-	49.2	-	49.9	-
18	42.5	2.93 dd (13.7, 4.1)	41.9	3.03 dd (14.4, 3.8)	41.8	3.03 dd (14.5, 3.6)
19	46.8	1.04, 2.27 t (13.6)	47.3	1.04, 2.28 t (13.4)	47.4	1.05 m, 2.28 t (13.5)
20	31.3	-	31.0	-	31.0	-
21	34.6	1.21 m, 1.39 m	36.1	1.15 m, 1.92	36.1	1.16 m, 1.92
22	33.1	1.56 m, 1.76	31.5	1.77, 1.91	31.6	1.77, 1.92
23	27.9	1.13 s	28.3	1.06 s	28.4	1.06 s
24	18.0	1.22 s	16.7	0.85 s	16.8	0.85 s
25	17.1	1.31 s	16.0	0.96 s	15.9	0.96 s
26	18.6	1.05 s	17.5	0.77 s	17.6	0.77 s
27	26.2	1.12 s	26.8	1.36 s	26.9	1.36 s
28	177.6	-	175.6	-	176.6	-
29	33.2	0.90 s	33.3	0.88 s	33.1	0.88 s
30	23.6	0.94 s	24.8	0.96 s	24.8	0.96 s

Overlapped signals are reported without multiplicity.

**Table 2:** ^13^C and ^1^H NMR spectroscopic data of the sugar moieties of **1**–**3** in CD_3_OD (*δ* in ppm, *J* in Hz).

Position	1		2		3	
	*δ*_C_	*δ*_H_	*δ*_C_	*δ*_H_	*δ*_C_	*δ*_H_
*3-O-*						
GlcA-1	106.6	4.37 d (7.8)	106.6	4.38 d (7.7)	106.7	4.43 d (7.7)
2	75.0	3.25 dd (9.3, 7.6)	75.1	3.24	74.6	3.29
3	77.3	3.36 t (9.0)	77.3	3.36 t (9.0)	80.8	3.70
4	72.9	3.51	72.9	3.51	74.5	3.96
5	76.2	3.51	75.9	3.77	73.6	3.53
6	172.3	-	171.3	-	171.7	-
Ara II-1					104.4	4.32 d (6.8)
2					72.1	3.55
3					73.7	3.52
4					69.4	3.82
5					67.2	3.59, 3.93
28-*O*-						
Ara I-1	93.6	5.64 d (4.0)	93.8	5.54 d (4.3)	93.8	5.54 d (4.3)
2	75.3	3.83	75.8	3.77	75.6	3.77
3	71.8	3.88	71.6	3.84	71.5	3.84
4	67.0	3.82	67.3	3.82	67.2	3.82
5	63.6	3.50, 3.91 dd (11.7, 7.5)	64.2	3.51, 3.89	64.1	3.51, 3.89 dd (12.5, 7.5)
Rha-1	101.1	5.10 br s	100.1	5.45 br s	100.1	5.45 br s
2	71.8	3.89 br s	72.0	3.92	71.9	3.92
3	71.8	3.87	72.6	3.60	72.6	3.61
4	83.1	3.56 t (9.1)	81.6	3.94	81.6	3.95
5	68.5	3.75	68.6	3.70	68.4	3.71
6	17.2	1.29 d (6.2)	18.1	1.28 d (6.3)	18.1	1.27 d (6.3)
Xyl-1	106.4	4.50 d (7.8)	106.9	4.41 d (7.7)	106.7	4.41 d (7.7)
2	75.7	3.21	82.6	3.58	82.5	3.58
3	77.8	3.31	74.5	3.48	74.6	3.48
4	70.7	3.47 m	70.0	3.80 m	70.0	3.80 m
5	66.9	3.18 t (10.9), 3.86	67.0	3.18 t (10.8), 3.86	66.9	3.18, 3.87
Glc-1			106.0	4.55 d (7.7)	106.0	4.55 d (7.7)
2			75.7	3.22	75.6	3.22 m
3			76.6	3.53	76.0	3.53
4			70.9	3.48	70.8	3.48
5			77.8	3.31 m	77.9	3.32 m
6			62.3	3.72, 3.78	62.4	3.72, 3.78

Overlapped signals are reported without multiplicity.

## Data Availability

Data is contained within the article or Supplementary Materials.

## Supplementary Materials

The following supporting information can be downloaded at: https://www.mdpi.com/article/10.3390/molecules29235781/s1, Figures S1–S6: spectra of compound **1**; Figures S7–S12: spectra of compound **2**; Figure S13–S18: spectra of compound **3**.
