# Supplementary material for: Three New Triterpene Glycosides from the Roots of Deutzia x Hybrida “Strawberry Fields” (Hydrangeaceae)
Source: Molecules. 2024 Dec 6;29(23):5781. doi: 10.3390/molecules29235781 (PMC11643241; doi:10.3390/molecules29235781)
Supplement: Supplementary file 1 [file molecules-29-05781-s001.zip › molecules-3287345-supplementary.pdf]

Supplementary Materials

Three New Triterpene Glycosides from the Roots of *Deutzia* x *Hybrida* "Strawberry Fields" (Hydrangeaceae)

Efstathia Karachaliou [1], David Pertuit [1], Antoine Bruguière [1], Marie-José Penouilh [2], Michel Picquet [2], Christine Belloir [1], Loïc Briand [1] and Anne-Claire Mitaine-Offer [1,*]

[1] Centre des Sciences du Goût et de l'Alimentation, CNRS, INRAE, Institut Agro, Université de Bourgogne, 21000 Dijon, CEDEX, France
[2] Institut de Chimie Moléculaire de l'Université de Bourgogne (UMR 6302), CNRS, Université de Bourgogne, 9 Av. Alain Savary, BP 47870, 21078 Dijon, CEDEX, France
* Correspondence: anne-claire.offer@u-bourgogne.fr

Contents

Figure S1. HSQC spectrum of compound **1** 3
Figure S2. HMBC spectrum of compound **1** 4
Figure S3. ROESY spectrum of compound **1** 5
Figure S4. ^{1}H spectrum of compound **1** 6
Figure S5. COSY spectrum of compound **1** 7
Figure S6. TOCSY spectrum of compound **1** 8
Figure S7. HSQC spectrum of compound **2** 9
Figure S8. HMBC spectrum of compound **2** 10
Figure S9. ROESY spectrum of compound **2** 11
Figure S10. ^{1}H spectrum of compound **2** 12
Figure S11. COSY spectrum of compound **2** 13
Figure S12. TOCSY spectrum of compound **2** 14
Figure S13. HSQC spectrum of compound **3** 15
Figure S14. HMBC spectrum of compound **3** 16
Figure S15. ROESY spectrum of compound **3** 17
Figure S16. ^{1}H spectrum of compound **3** 18
Figure S17. COSY spectrum of compound **3** 19
Figure S18. TOCSY spectrum of compound **3** 20

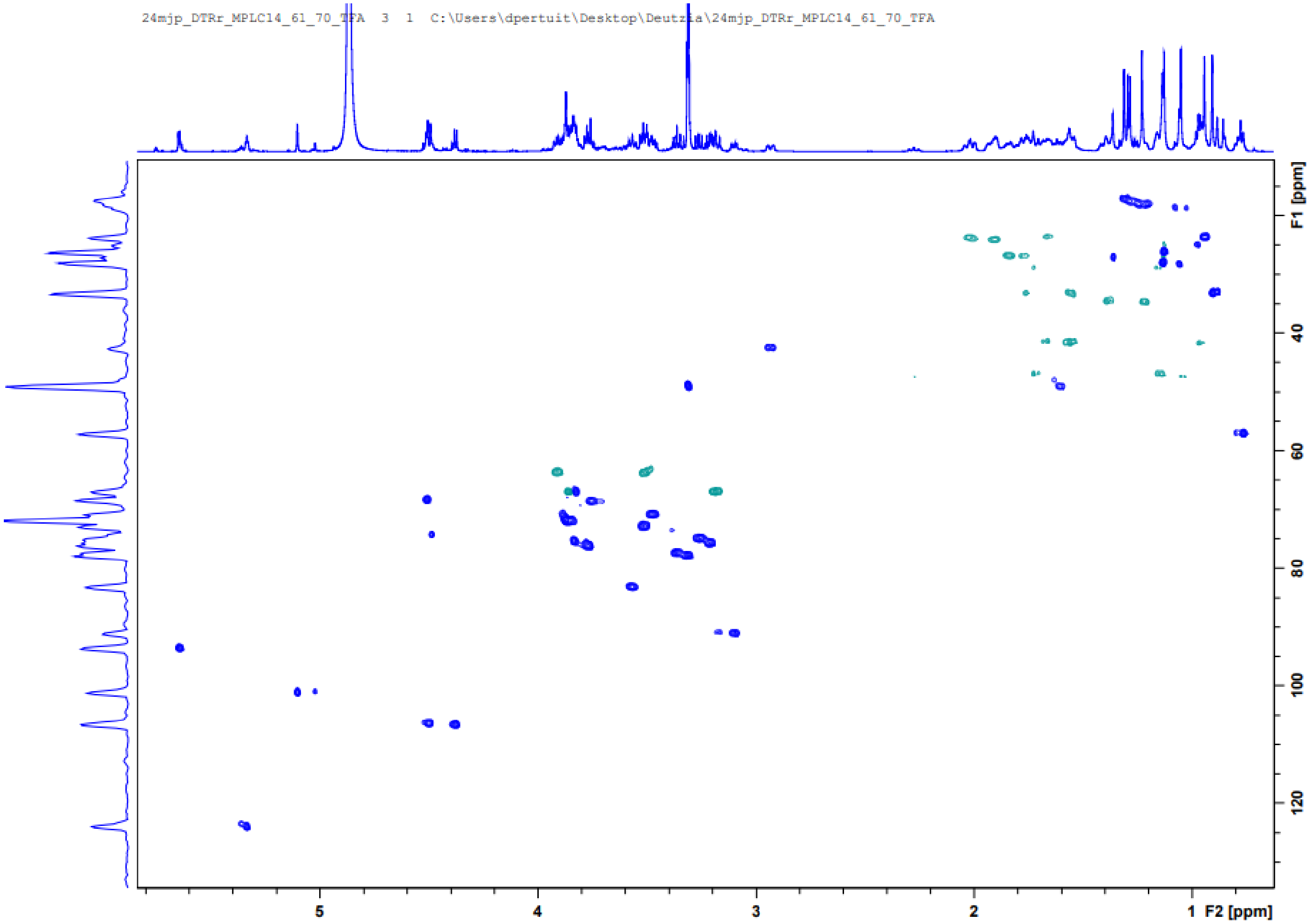

Figure S1. HSQC spectrum of compound **1**

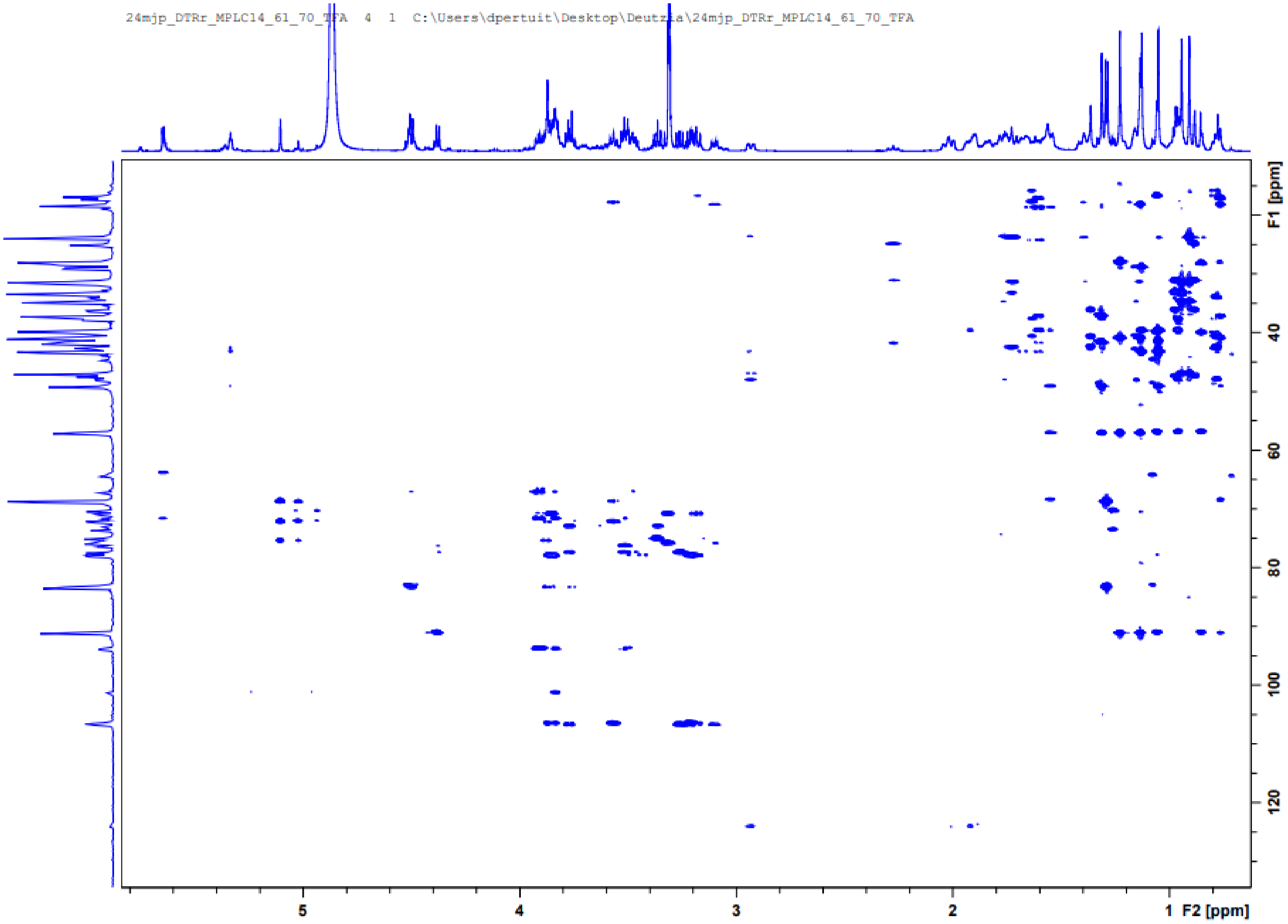

Figure S2. HMBC spectrum of compound **1**

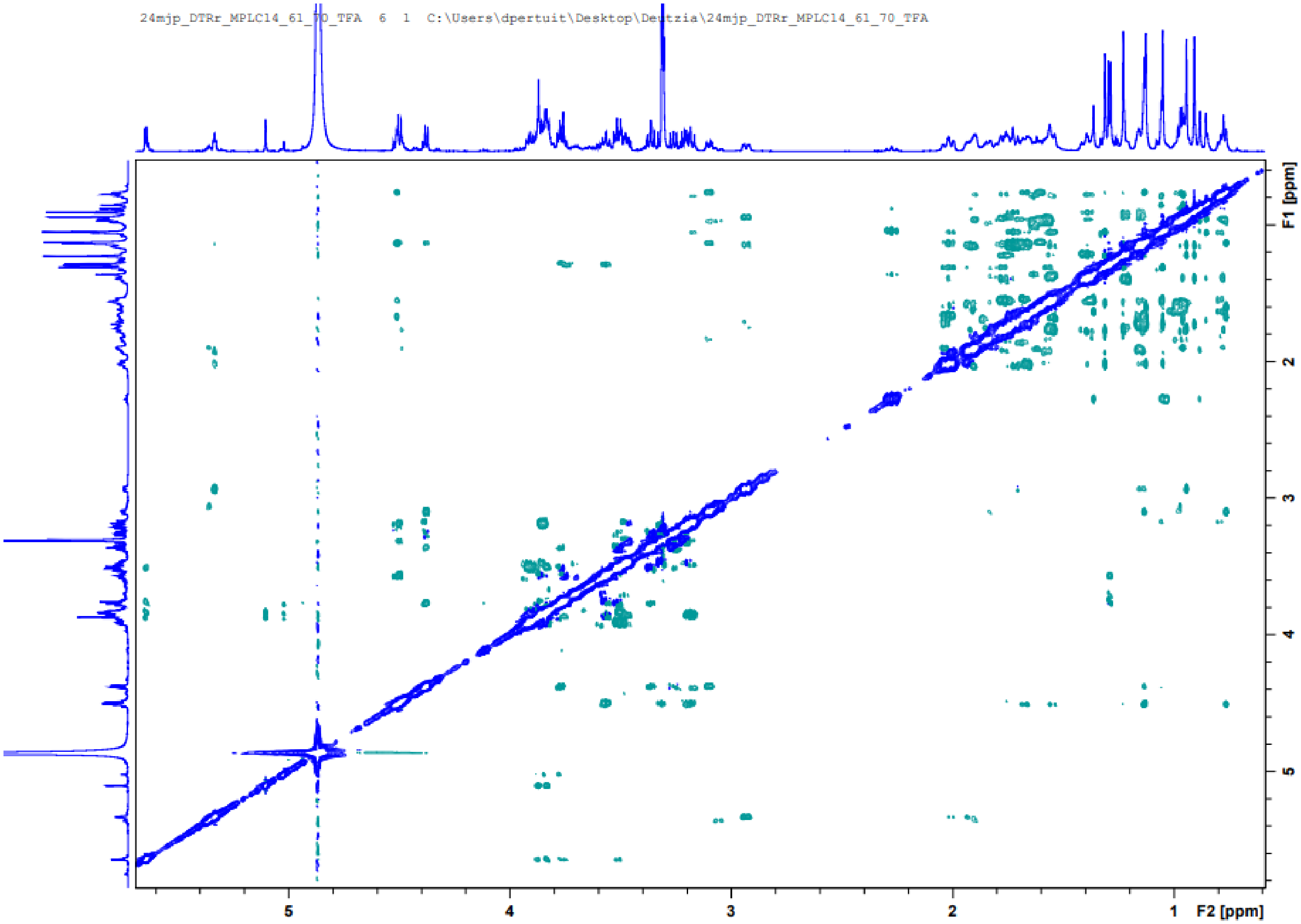

Figure S3. ROESY spectrum of compound **1**

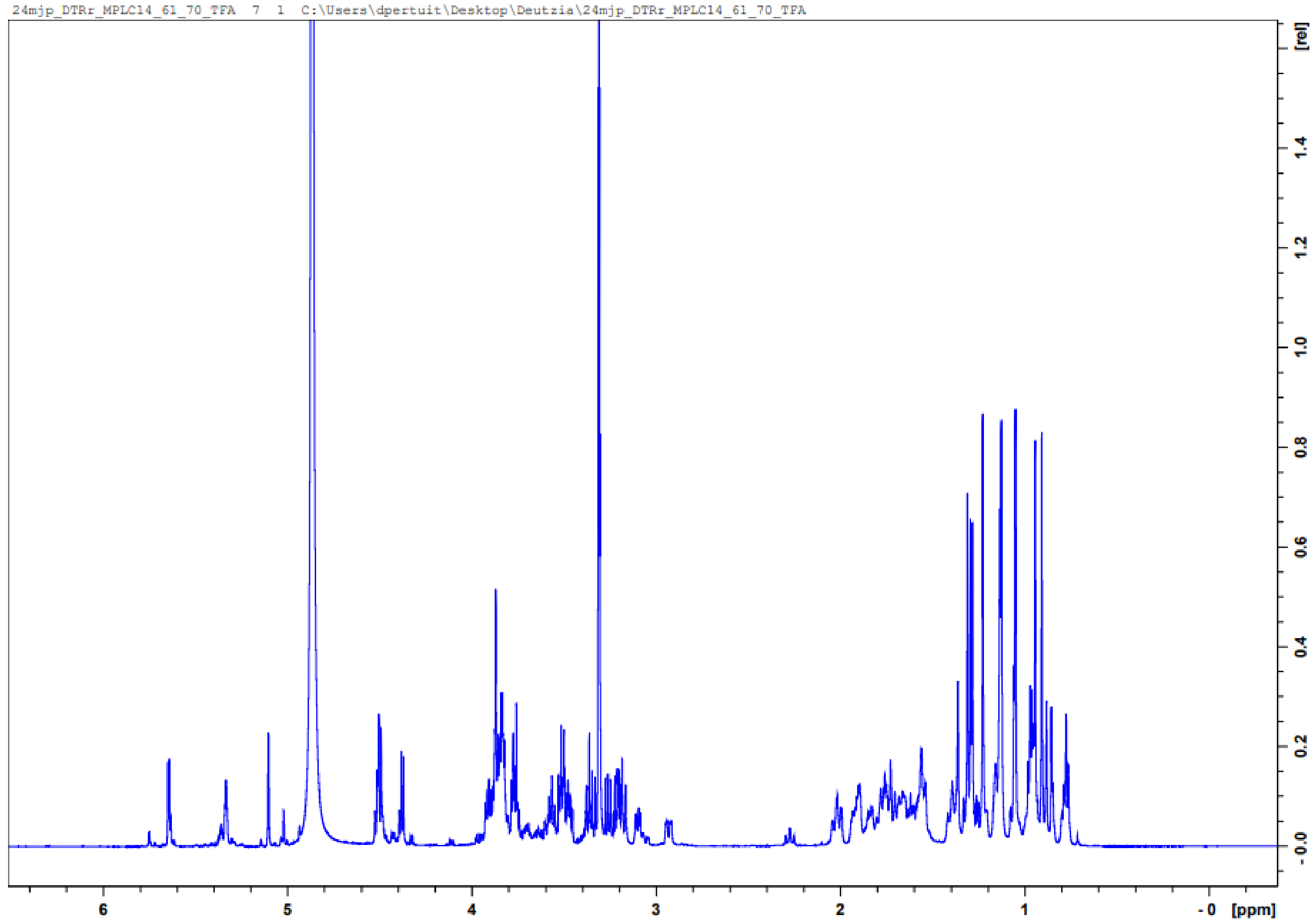

Figure S4. ^{1}H spectrum of compound **1**

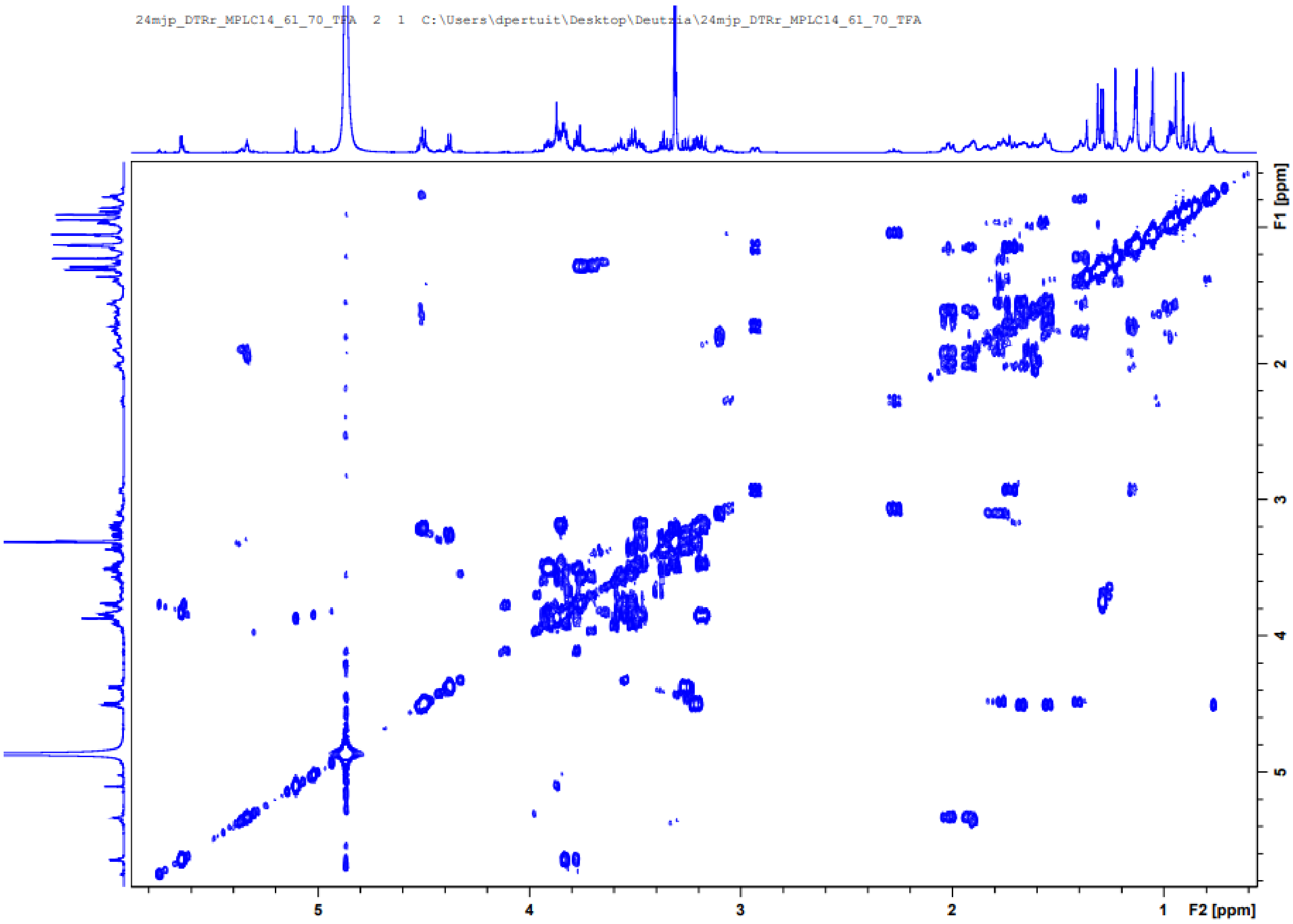

Figure S5. COSY spectrum of compound **1**

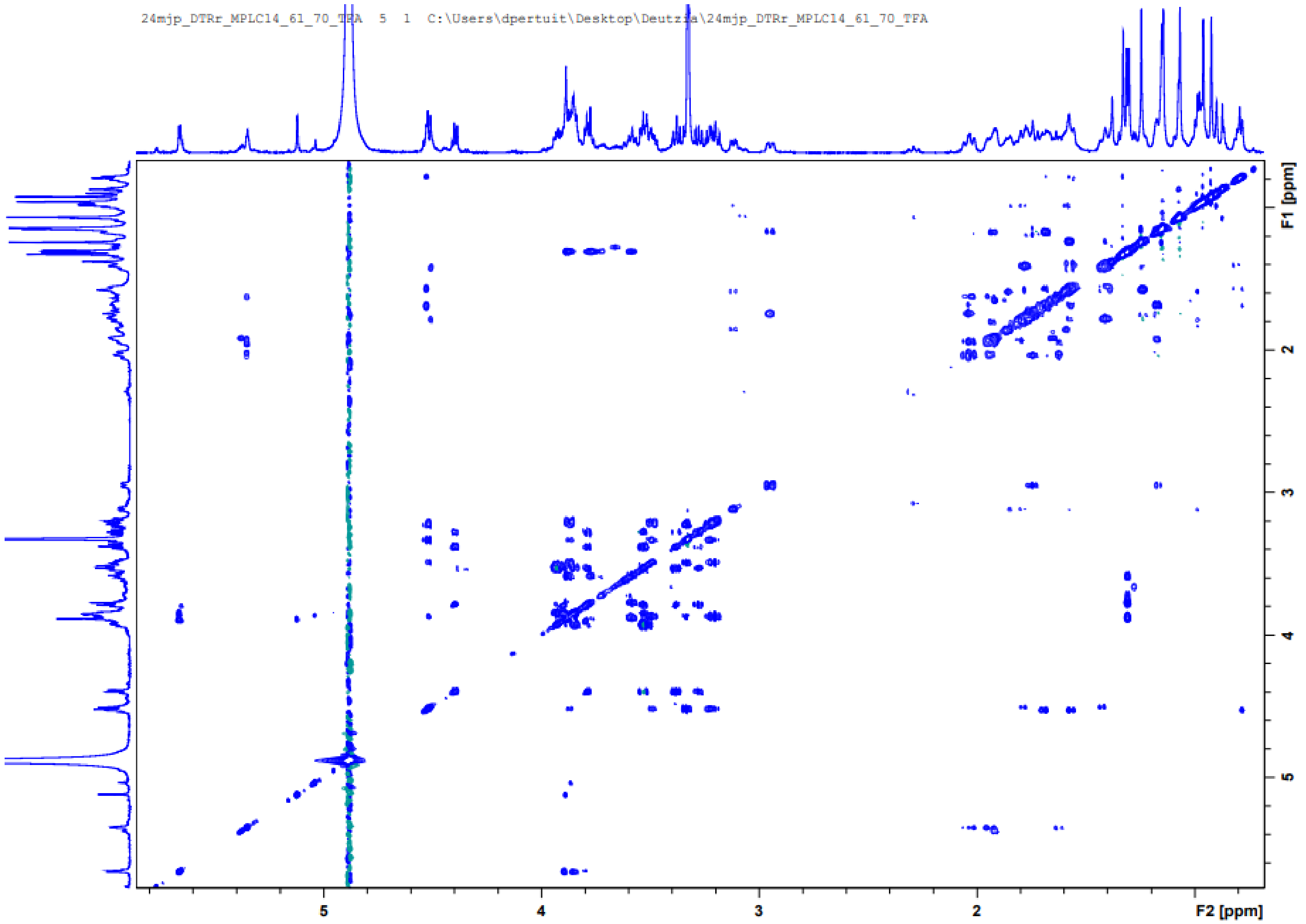

Figure S6. TOCSY spectrum of compound **1**

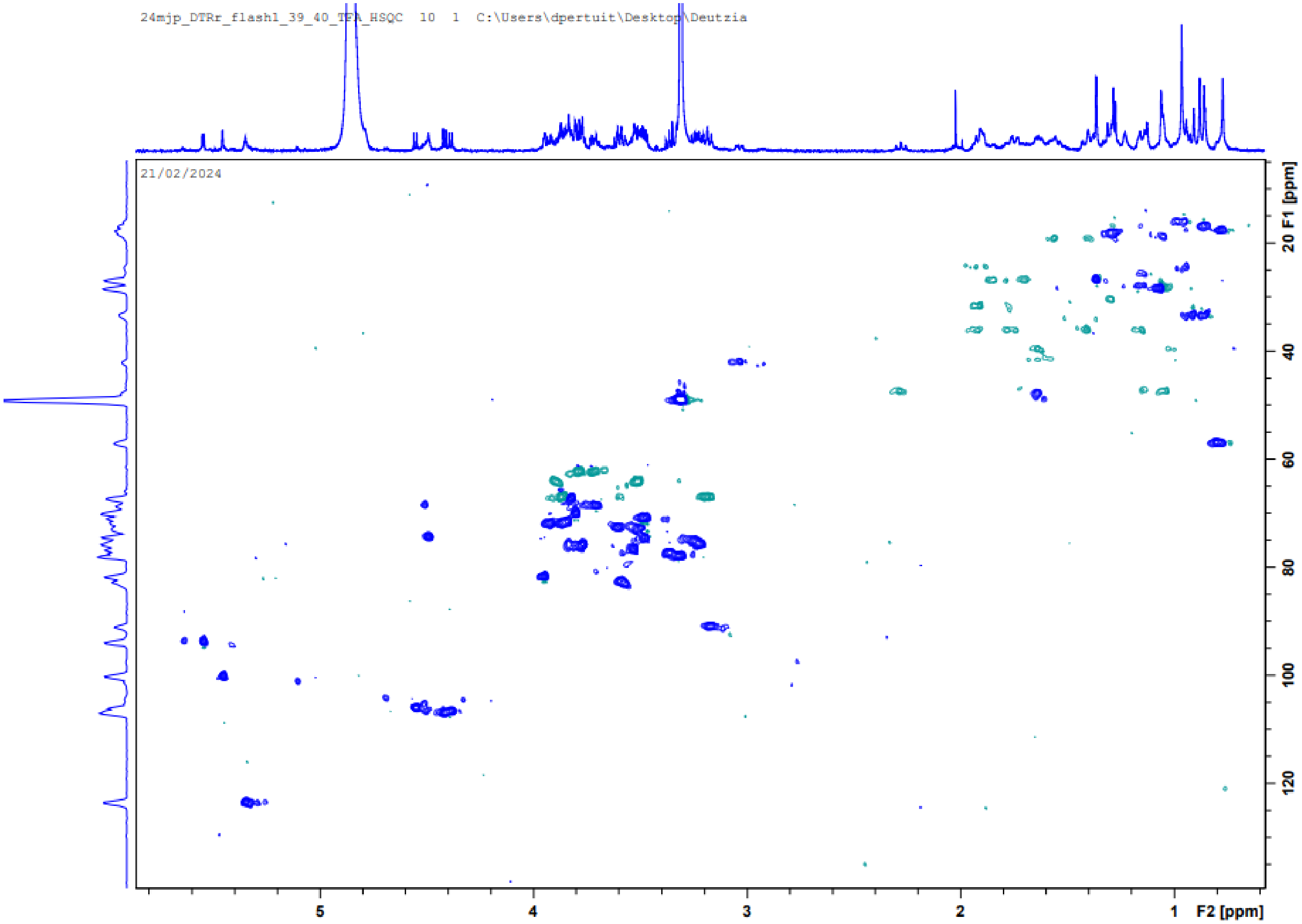

Figure S7. HSQC spectrum of compound **2**

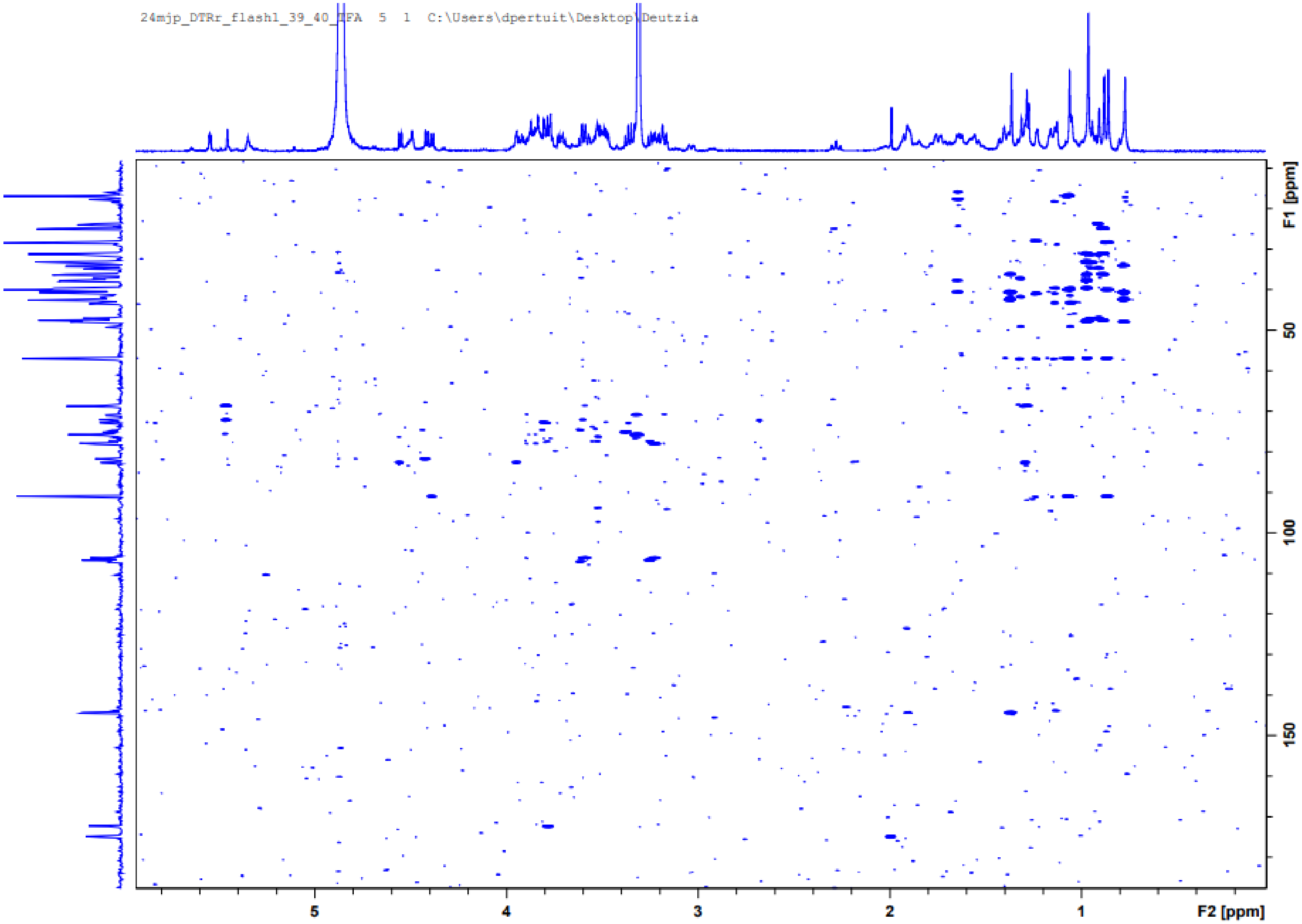

Figure S8. HMBC spectrum of compound **2**

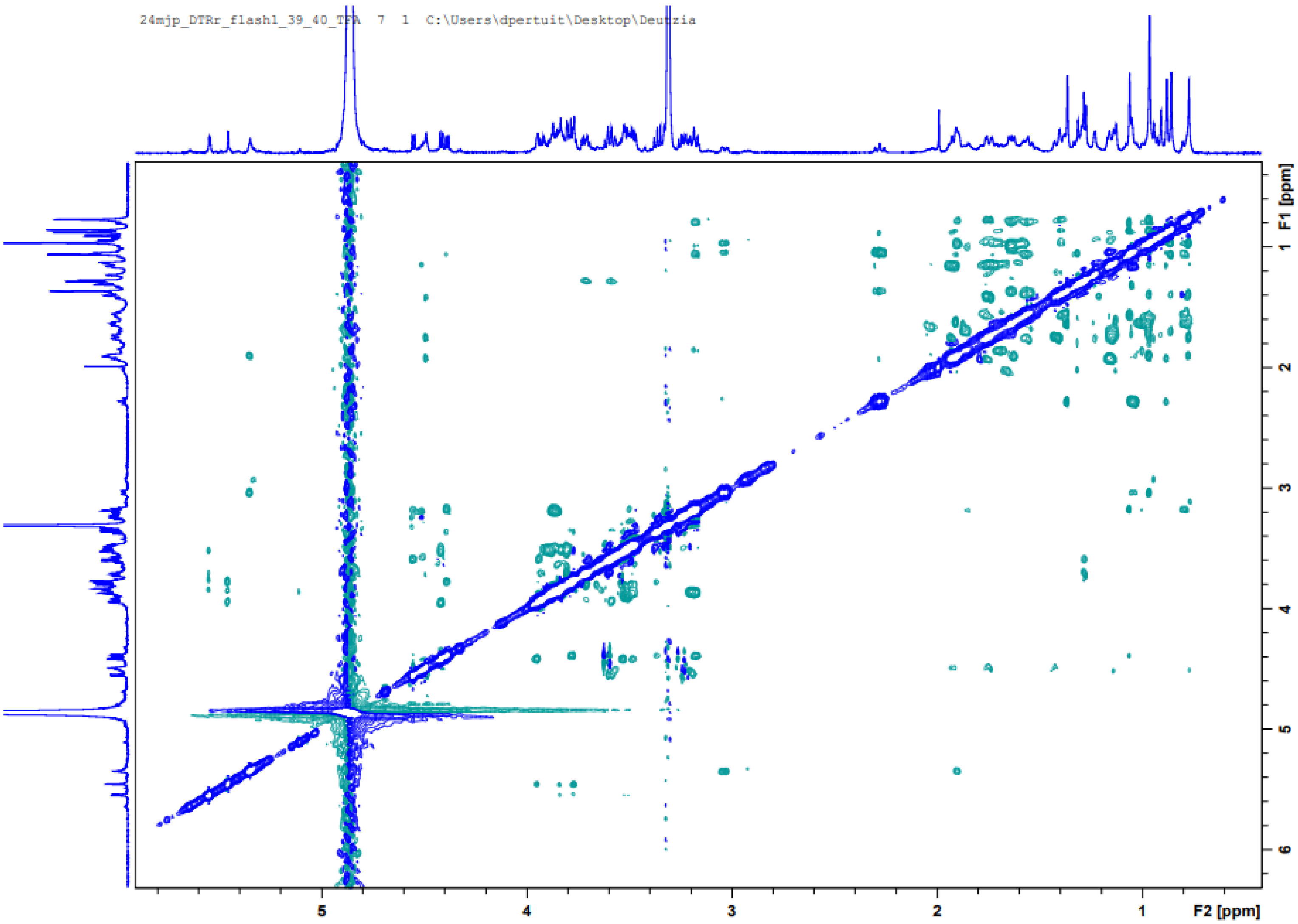

Figure S9. ROESY spectrum of compound **2**

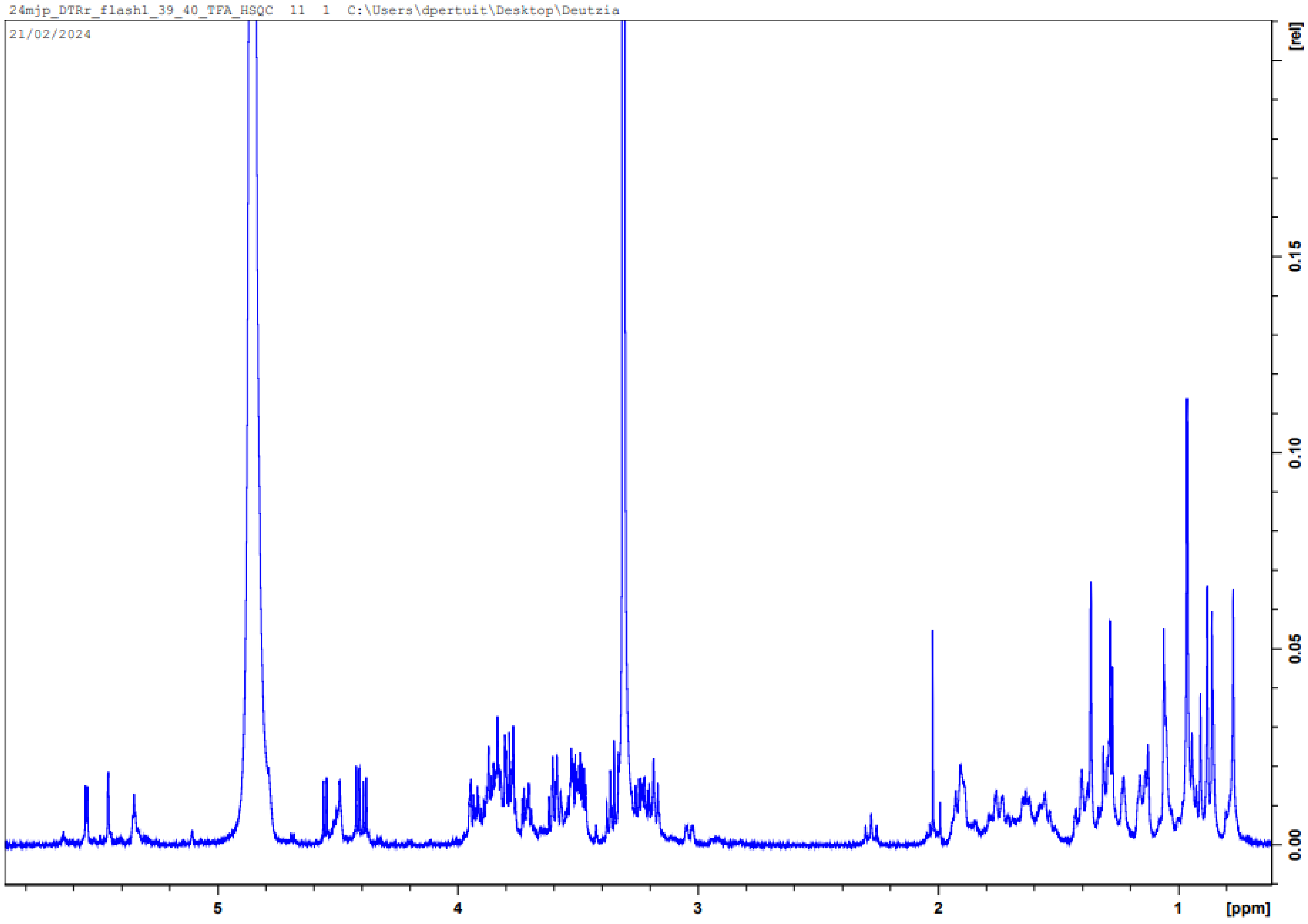

Figure S10. ^{1}H spectrum of compound **2**

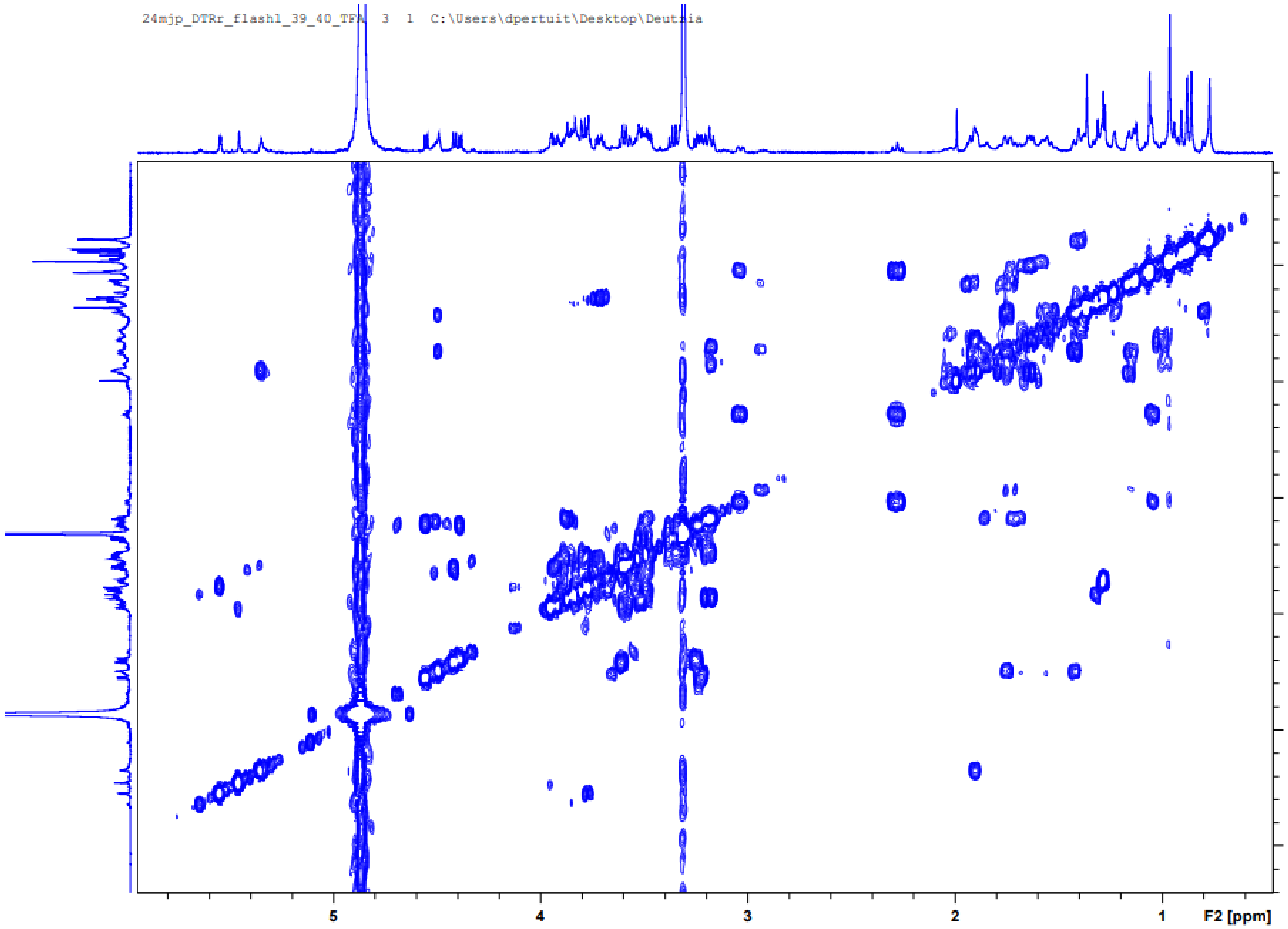

Figure S11. COSY spectrum of compound **2**

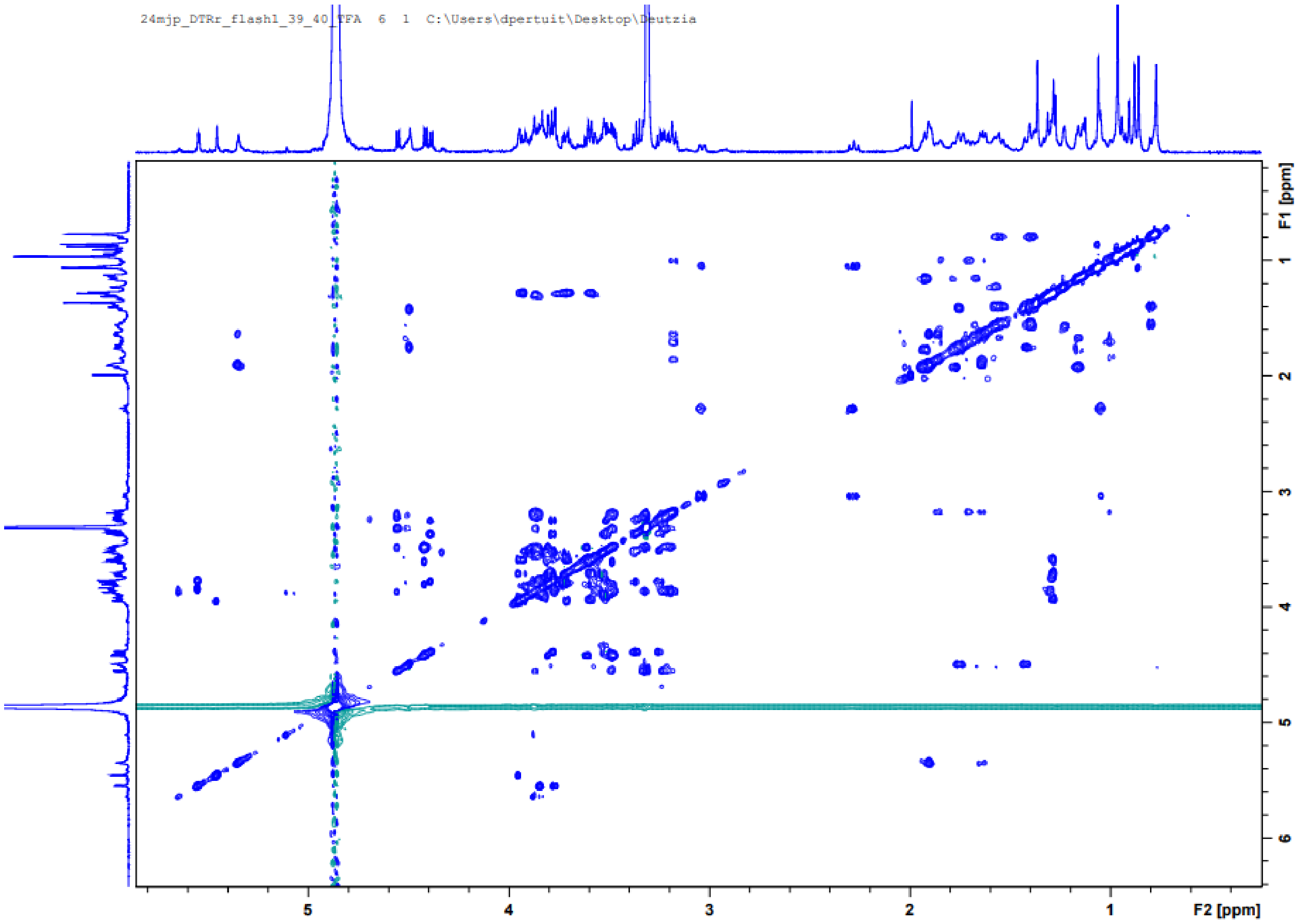

Figure S12. TOCSY spectrum of compound **2**

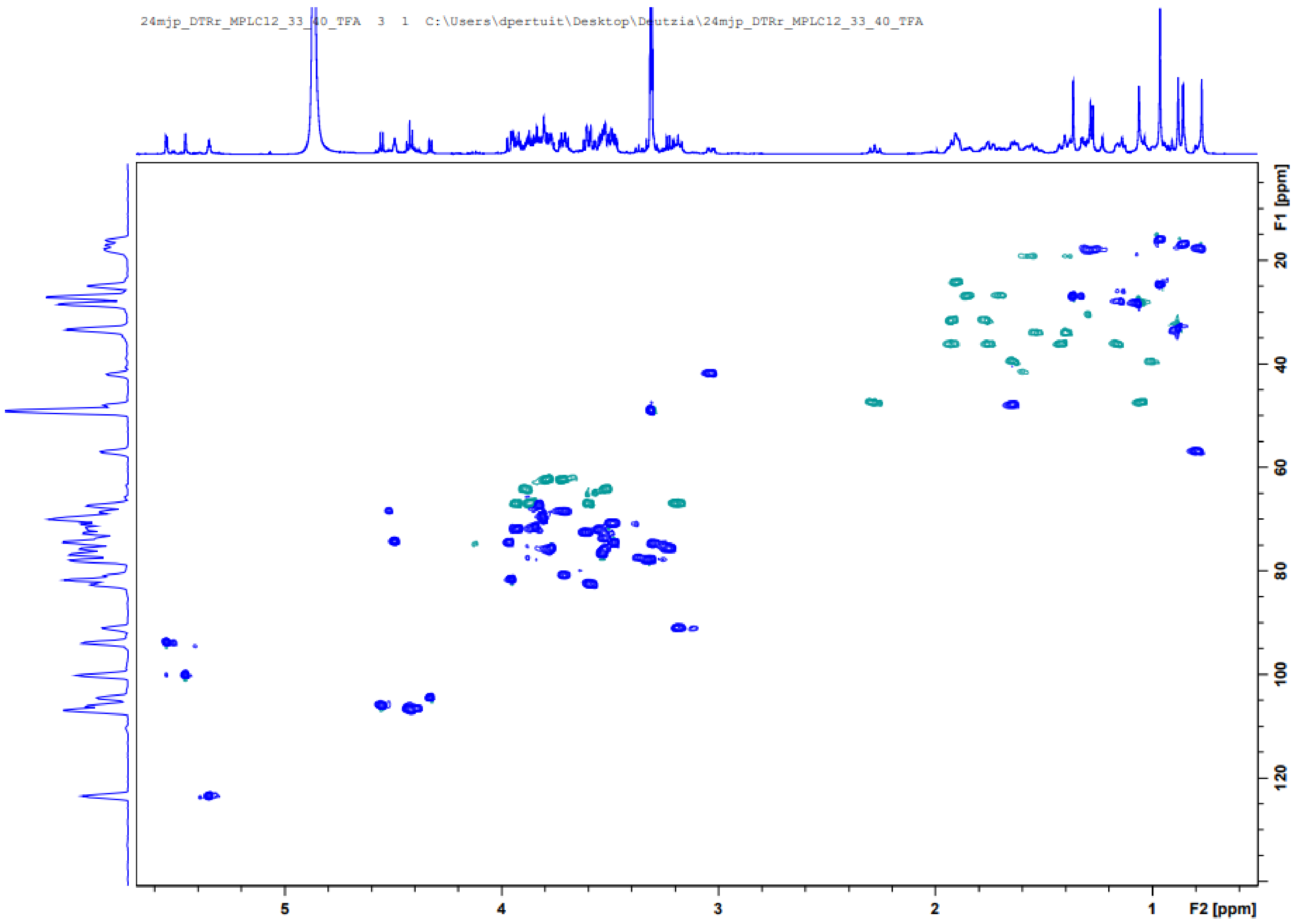

Figure S13. HSQC spectrum of compound **3**

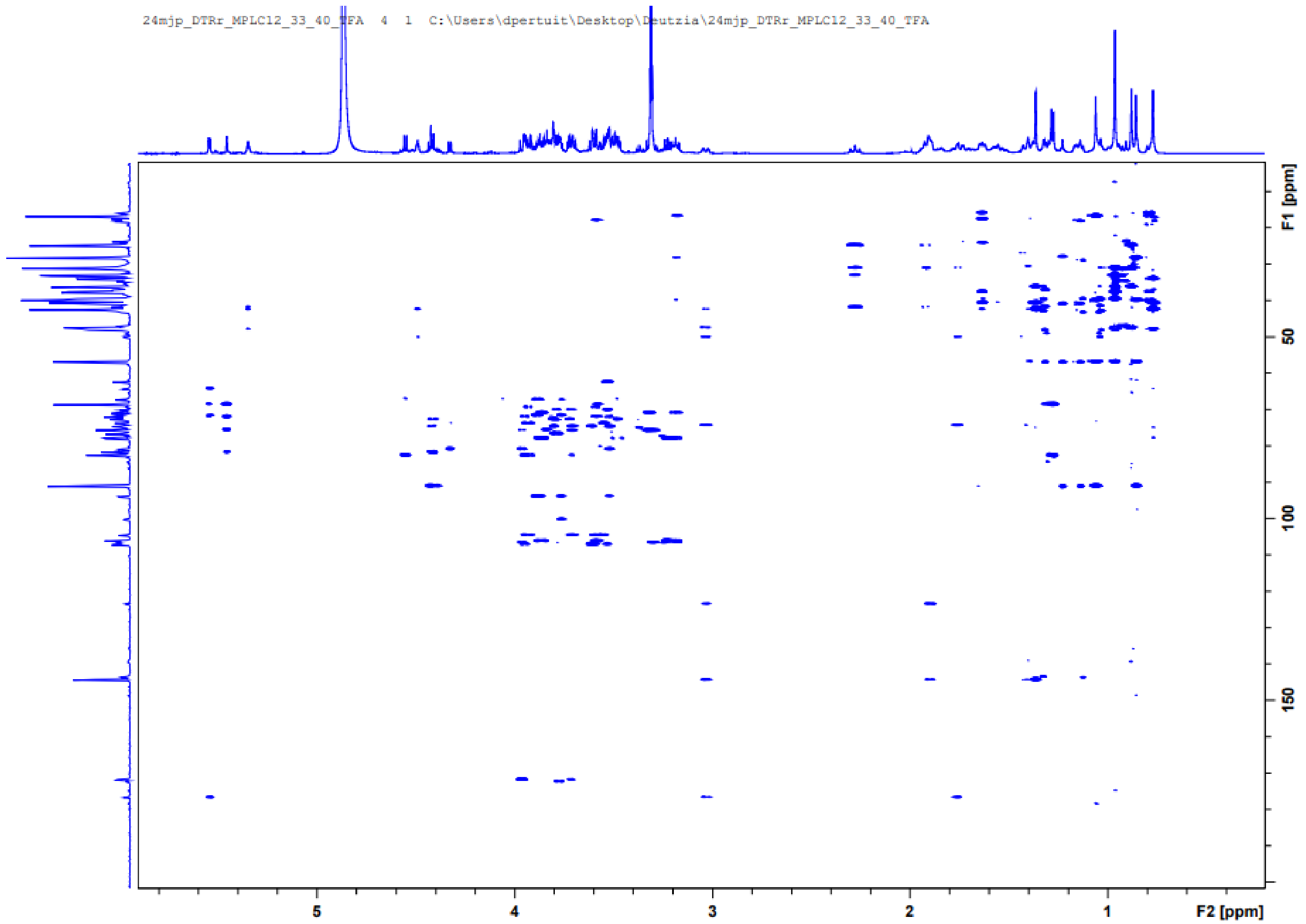

Figure S14. HMBC spectrum of compound **3**

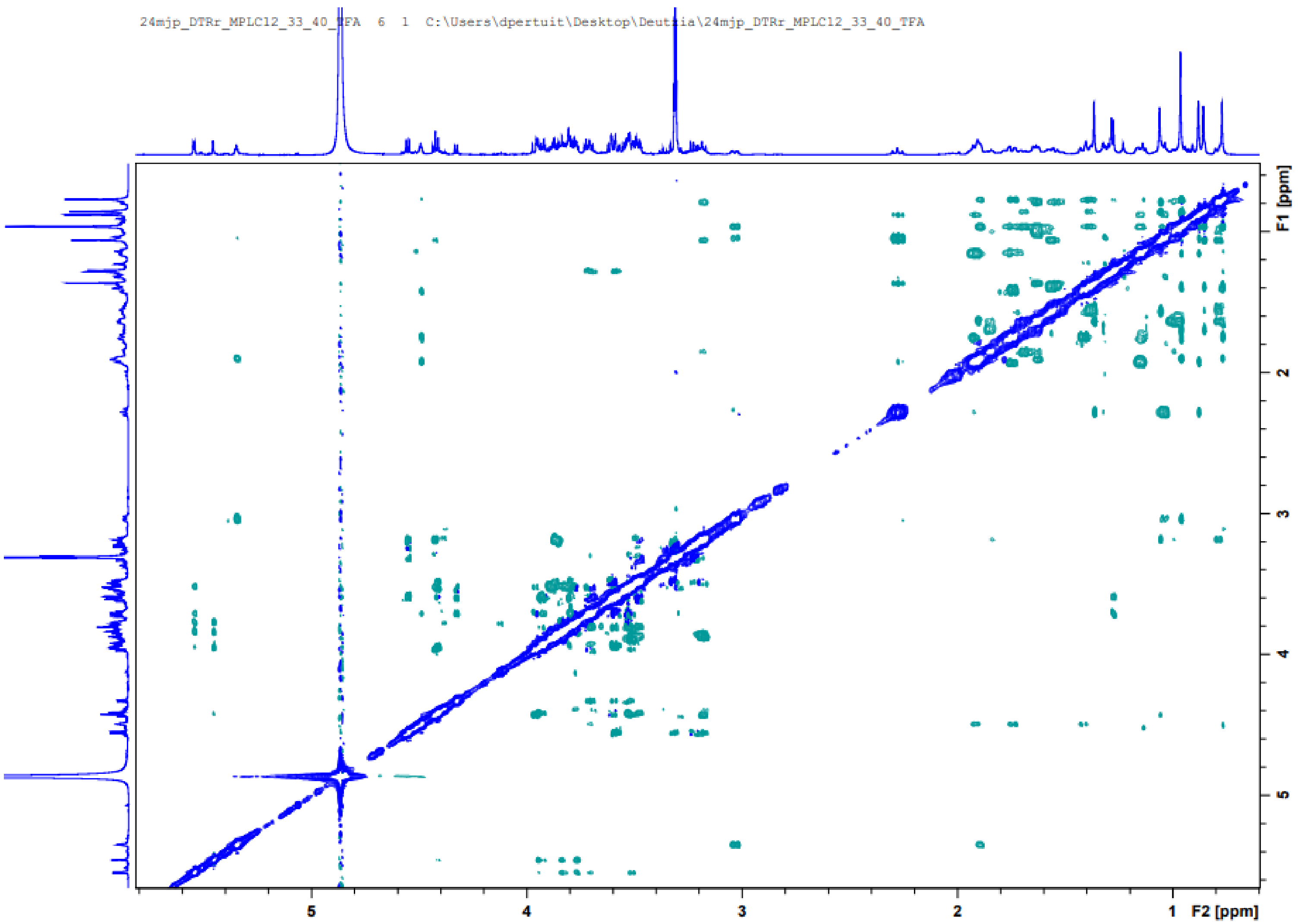

Figure S15. ROESY spectrum of compound **3**

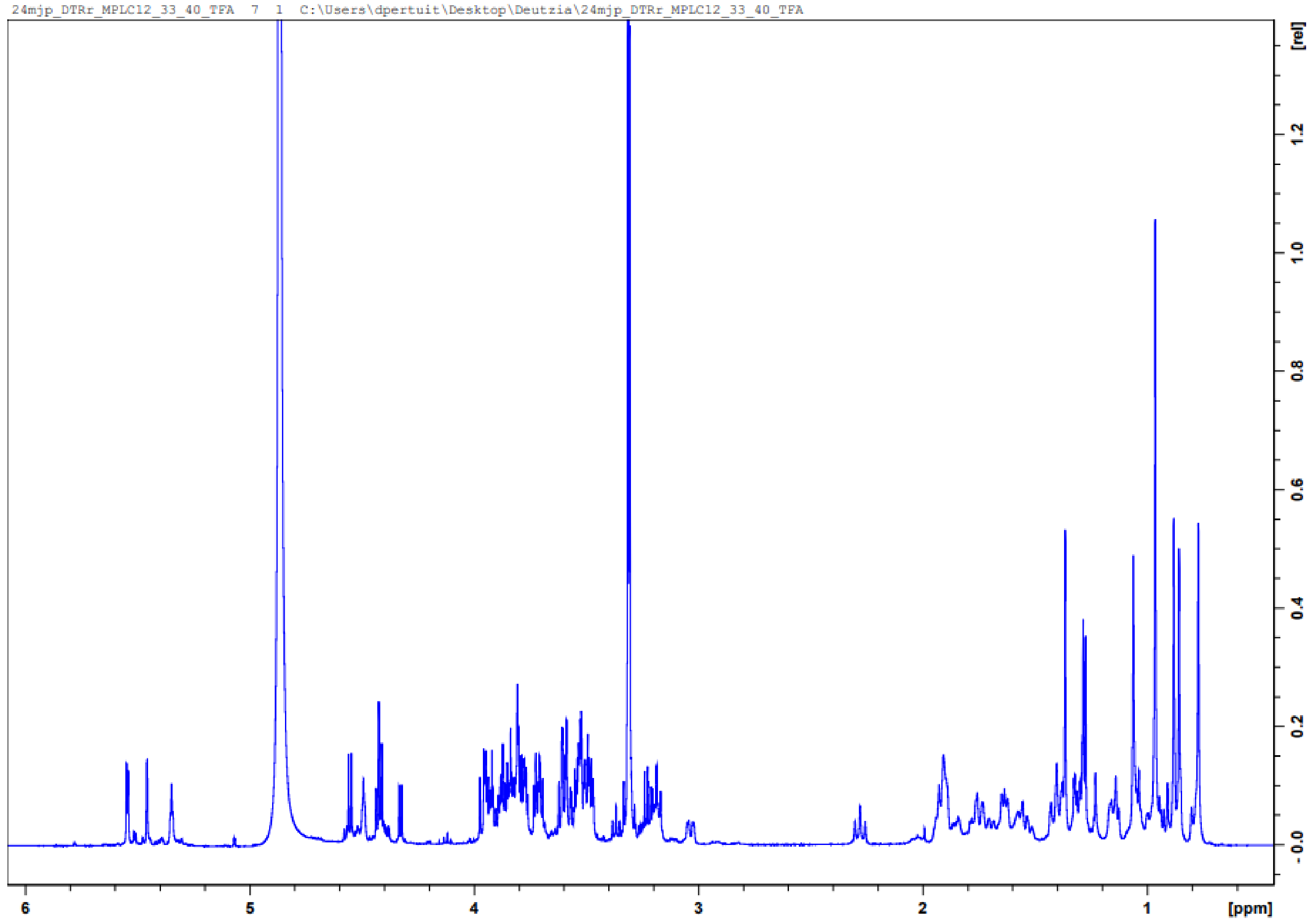

Figure S16. ^{1}H spectrum of compound **3**

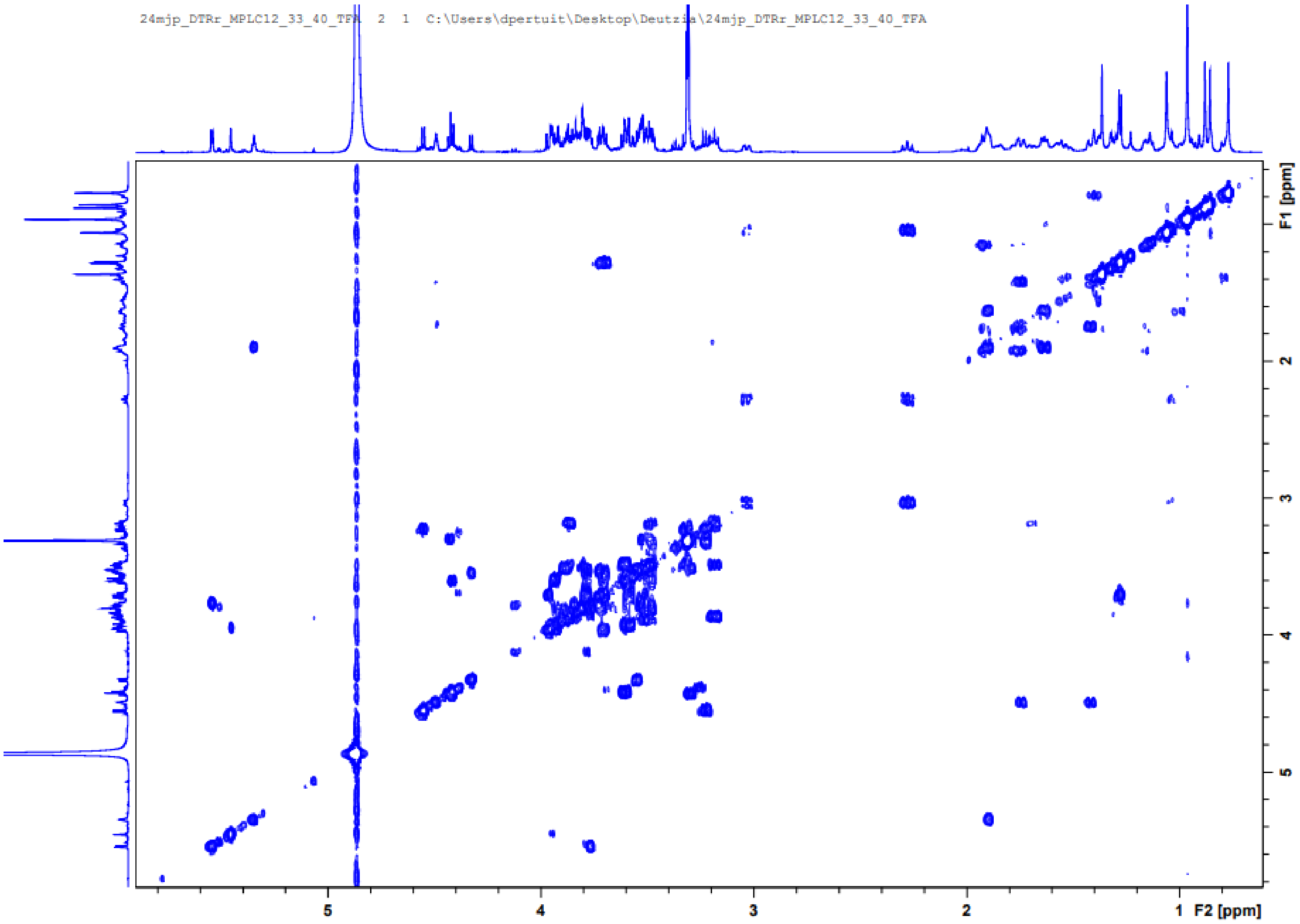

Figure S17. COSY spectrum of compound **3**

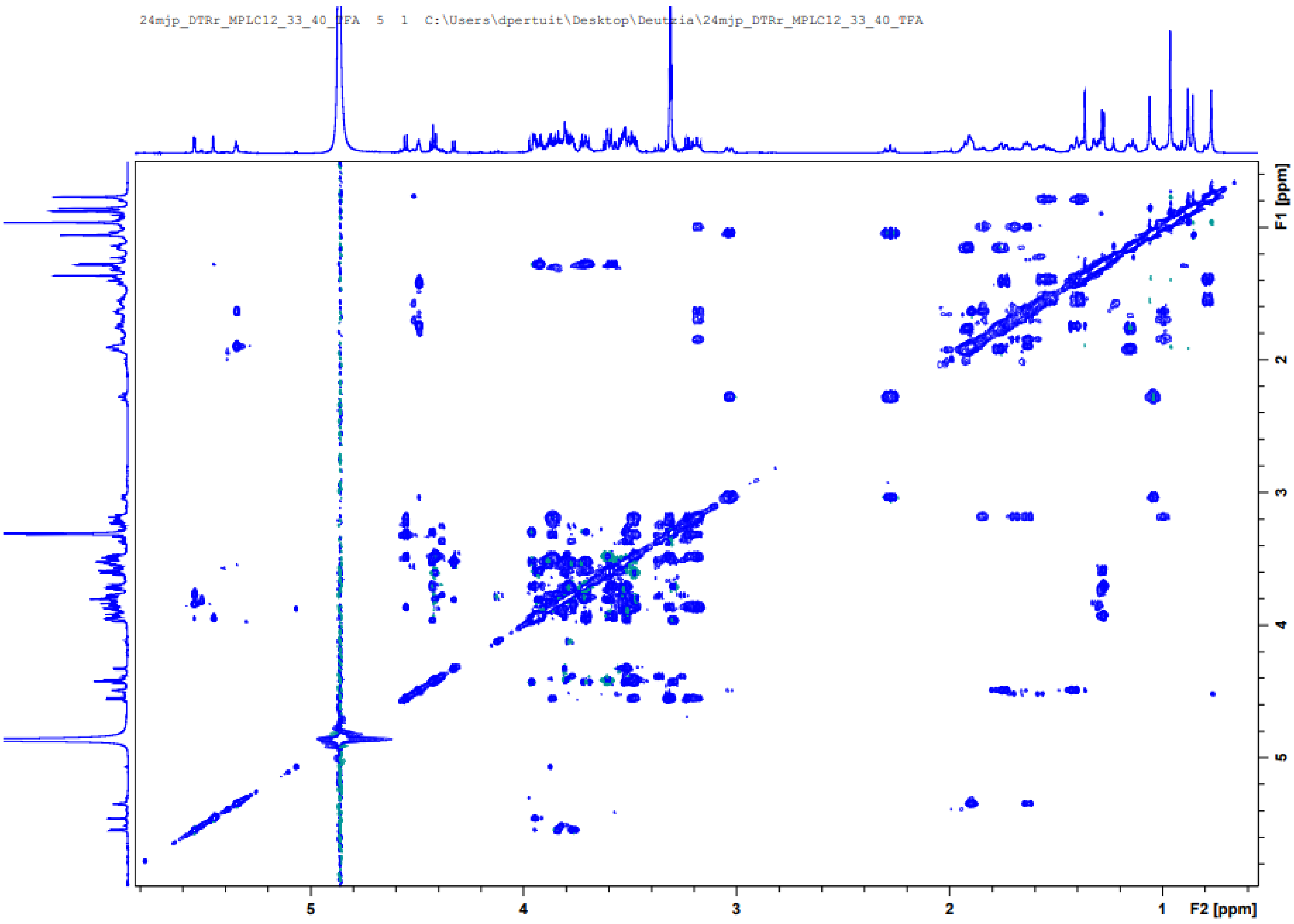

Figure S18. TOCSY spectrum of compound **3**
